# An anoikis-related signature predicts prognosis and immunotherapy response in gastrointestinal cancers

**DOI:** 10.3389/fimmu.2025.1477913

**Published:** 2025-02-06

**Authors:** Ruyi Liu, Yuchen Liu, Weicheng Huang, Pengxiang Chen, Yufeng Cheng

**Affiliations:** ^1^ Department of Radiation Oncology, Qilu Hospital of Shandong University, Jinan, China; ^2^ Shandong Provincial Key Laboratory of Malignant Tumor Precision Treatment, Jinan, China; ^3^ Shandong Provincial Engineering Research Center for Tumor Precision Treatment, Jinan, China; ^4^ Cancer Institute of Shandong University, Jinan, China; ^5^ Neutron Medical Center, Qilu Hospital of Shandong University, Jinan, China

**Keywords:** gastrointestinal cancers, anoikis, prognostic prediction, immunotherapy, tumor immune microenvironment

## Abstract

**Background:**

Gastrointestinal (GI) cancers have high incidence rates and mortality rates. Anoikis is a special type of cell apoptosis, and anoikis resistance has been reported to be associated with tumor malignancy. We aimed to explore the roles of anoikis-related genes (ARGs) in the GI cancer prognosis.

**Methods:**

We extracted RNA sequencing and clinical data from The Cancer Genome Atlas and Gene Expression Omnibu databases for patients with esophageal cancer, gastric cancer, colon cancer and rectal cancer and identified ARGs from GeneCards and Harmonizome. Anoikis-related patterns were identified via unsupervised clustering analysis. We constructed a prognostic signature (Anoscore) based on prognostic ARGs through univariate, LASSO, and multivariate Cox regression analyses. The model was validated and evaluated using Kaplan–Meier analysis, receiver operating characteristic curves, univariate Cox regression analysis, multivariate Cox regression analysis, column charts, and calibration curves. We also performed a single-cell sequencing analysis of candidate genes via TISCH2. A correlation analysis between the Anoscore, the tumor microenvironment and drug sensitivity was conducted in GI cancers. The expression and function of some candidate genes were validated *in vitro*.

**Results:**

In terms of prognostic ARGs, two anoikis-related patterns, ARG clusters A and B, were identified. ARG cluster B had a worse prognosis than did ARG cluster A. Subsequently, the Anoscore was developed as an independent prognostic factor. It demonstrated the robust predictive capability for the prognosis of patients with GI cancers. Notably, patients with high Anoscores exhibited poor outcomes. In addition, we established a nomogram (Ano-nomogram) based on the Anoscore and clinicopathological factors of patients to predict the 3-year and 5-year survival probabilities. Moreover, patients with high Anoscores had higher levels of immune cell infiltration and higher immune checkpoint expression. The drug sensitivity analysis revealed that patients with high or low Anoscores were sensitive to different chemotherapies and targeted drugs. S100A11 and TLR3, representative candidate genes, exhibited different expression patterns and biological functions.

**Conclusion:**

This study highlighted the significant potential of the Anoscore in predicting prognosis and guiding the selection of personalized therapeutic regimens for patients with GI cancers.

## 1 Introduction

Malignant tumors are diseases that seriously endanger human health worldwide. According to data from Global Cancer Statistics 2022 (1), gastrointestinal (GI) cancers, including esophageal cancer (EC), gastric cancer (GC), colon cancer (CC) and rectal cancer (RC), account for almost 20% of the 20.0 million new cases and 20.6% of the 9.7 million deaths worldwide. The incidence rates of EC, GC and colorectal cancer (CRC) ranked 11th, 5th, and 3rd, respectively, while the mortality rates ranked 7th, 5th, and 2nd, respectively. At present, comprehensive strategies (including surgery, radiotherapy, chemotherapy, targeted therapy and immunotherapy) have been applied to GI cancers. However, the prognosis of patients with GI cancers is unsatisfactory, and effective prognostic indicators are still lacking. Therefore, exploring promising prognostic biomarkers to identify high-risk patients with GI cancers and apply individual treatments remains an urgent task.

At present, many prognostic prediction systems, such as mRNA risk models, have been established to predict the tumor prognosis. For example, Huang et al. (2) developed a cuproptosis-related signature for CRC with AUCs of 0.777 and 0.768 at 3 and 5 years, respectively. For EC, Ren et al. (3) reported a fibroblast-associated mRNA risk score with AUC values of 0.73, 0.76 and 0.78 at 1, 2, and 3 years, respectively. A metabolism-related mRNA risk signature for GC constructed by Liu et al. had AUC values of 0.700, 0.700 and 0.640 at 1, 3 and 5 years, respectively (4). These prognostic prediction systems, which are based on mRNAs, have displayed outstanding accuracy and reliability, suggesting that developing effective mRNA prognostic signatures is highly valuable. However, universal biomarkers that are effective for EC, GC and CRC, which are highly beneficial for clinical applications, are lacking.

Anoikis is a special type of cell apoptosis that initiates when cells detach from the neighboring cellular or extracellular matrix (ECM), which plays an essential role in cellular homeostasis, proliferation and differentiation (5). When the connections between normal cells and adjacent cells or the ECM are disrupted, anoikis occurs and results in cell death (6). However, cancer cells can acquire resistance to anoikis, which can prevent cell death and maintain cell proliferation. Research has reported that tumor cells with anoikis resistance can proliferate in distant areas after detaching from nests. It is one of the important mechanisms by which tumor cells enhance invasion and metastasis (6). Research has also shown that anoikis resistance assists in immune escape, alters the tumor microenvironment (TME) and induces chemotherapy resistance (7). Although anoikis plays a crucial role in the progression and metastasis of various solid tumors, systematic investigations of anoikis-related genes (ARGs) in GI cancers are limited. Therefore, we aimed to establish an anoikis-associated risk signature for predicting the prognosis of patients with GI cancers.

Here, we identified prognostic ARGs in GI cancers and recognized two anoikis subtypes. An original risk model named the Anoscore, which can predict clinical outcomes effectively, was subsequently constructed and verified. We also found that the Anoscore could predict drug sensitivity and was highly related to immune cell infiltration in GI cancers. Taken together, we identified ARGs and constructed an Anoscore related to the prognosis and the tumor immune microenvironment (TIME), which contributed innovative viewpoints for the prediction of the prognosis and precise treatment of GI cancers.

## 2 Materials and methods

### 2.1 Data and resources

RNA-sequencing (RNA-seq) data and complete clinical information for esophageal carcinoma (ESCA), stomach adenocarcinoma (STAD), colon adenocarcinoma (COAD) and rectum adenocarcinoma (READ) samples from a total of 1255 patients were extracted from The Cancer Genome Atlas (TCGA) official website (https://portal.gdc.cancer.gov/). The RNA-seq data were downloaded as Fragments Per Kilobase of transcript per Million mapped reads (FPKM) values and log2-transformed for subsequent analysis. Additionally, we obtained the RNA-seq data and clinical data of 483 GC patients in the GSE84437 dataset and 585 CRC patients in the GSE39582 dataset from the Gene Expression Omnibus (GEO) database (https://www.ncbi.nlm.nih.gov/gds). Furthermore, 647 ARGs in total were acquired from GeneCards (https://www.genecards.org/) and Harmonizome (https://maayanlab.cloud/Harmonizome/).

### 2.2 Anoikis-related patterns

The differential expression analysis between normal and tumor samples was performed using Wilcoxon rank-sum test. The criteria for identifying differentially expressed ARGs were set as |log2FC| > 1 and false discovery rate (FDR) < 0.05. Gene expression values were normalized prior to analysis. Univariate Cox proportional hazards regression analysis was conducted to identify ARGs significantly associated with overall survival (OS) (P < 0.05). In accordance with the expression profiles of these ARGs, the R package ‘ConsensusClusterPlus’ was utilized to perform clustering and identify anoikis-related patterns. The consensus matrix was used to acquire the optimal number of clusters. Principal component analysis (PCA), t-distributed stochastic neighbor embedding (t-SNE) and uniform manifold approximation and projection (UMAP) were adopted to evaluate the reliability of anoikis-related patterns. Kaplan-Meier (KM) curves were generated to evaluate the prognostic differences between clusters. Single-sample gene set enrichment analysis (ssGSEA) was applied to obtain the immune cell infiltration abundance. Gene set variation analysis (GSVA) and gene set enrichment analysis (GSEA) were used to conduct Kyoto Encyclopedia of Genes and Genomes (KEGG) enrichment analysis, characterizing the functions and pathways of genes in different clusters.

### 2.3 Establishment of a risk score model (Anoscore) based on ARGs

All GI patients (n = 2220) from the TCGA and GEO databases were randomly divided into two equal-sized cohorts using the “caret” R package: a training cohort (n = 1110) and a test cohort (n = 1110). The training cohort was utilized to develop the risk score model, named Anoscore, while the test cohort was used for internal validation. To ensure the robustness of our validation process, we maintained similar distributions of key clinical characteristics between the two cohorts through stratified random sampling. The training cohort was utilized to develop Anoscore through a two-step selection process. First, least absolute shrinkage and selection operator (LASSO) regression was applied to reduce dimensionality and select the most significant candidate genes among the ARGs associated with OS. Multivariate Cox regression analysis was then performed on the LASSO-selected genes to further refine the model. The Anoscore was calculated using the following formula: Anoscore = Σ in(Coefi * Xi). X represents the expression level of each gene, and Coefi represents the weighted coefficient of the corresponding gene. The accuracy of the Anoscore in the training cohort was evaluated using KM and receiver operating characteristic (ROC) curves.

Subsequently, the performance of Anoscore was evaluated in the test cohort through multiple approaches for internal validation. KM and ROC curves were plotted to validate the accuracy of the prognostic signature in the test cohort. Moreover, the independent prognostic value of the Anoscore was determined via multivariate Cox regression analysis. Furthermore, the associations among cluster A, cluster B, the Anoscore and patient survival were analyzed and visually represented.

### 2.4 Construction and assessment of a predictive nomogram (Ano-nomogram)

An Ano-nomogram was constructed using the ‘rms’ R package to predict OS by integrating Anoscore with key clinical features, including age, sex, and pathologic N stage, in GI tumor patients. The Cox proportional hazards regression model was used as the basis for the nomogram construction. The performance of the Ano-nomogram was evaluated through various validation methods. Calibration curves were generated to assess the agreement between predicted and observed survival probabilities at 1, 3, and 5 years. Additionally, decision curve analysis (DCA) was performed to evaluate the clinical utility of the Ano-nomogram compared with individual predictors, including Anoscore, age, gender, T stage, and N stage, at different time points. Cumulative hazard curves were also generated by stratifying patients into high- and low-risk groups based on Ano-nomogram scores to visualize the model’s discriminative ability.

### 2.5 Single-cell sequencing analysis

The tumor immune single-cell hub 2 (TISCH2) database (http://tisch.comp-genomics.org/), which provides detailed cell type annotations at the single-cell level, was used to analyze the expression of S100A11 and TLR3 at the single-cell level. The correlations between the expression of genes and infiltrating immune cells were also assessed, enabling us to explore the TME in detail.

### 2.6 Evaluation of drug sensitivity

Drug response information and information on drug targeting pathways were obtained from the Genomics of Drug Sensitivity in Cancer (GDSC) database (https://www.cancerrxgene.org/). Drug sensitivity was predicted using the oncoPredict algorithm with empirical Bayes batch correction. Correlation analyses were performed between the Anoscore and the predicted drug sensitivity to identify potential therapeutic implications.

### 2.7 Immune landscape based on the Anoscore

The TME of GI cancers was comprehensively characterized. The XCELL, TIMER, QUANTISEQ, MCPOUNTER, EPIC, CIBERSORT-ABS and CIBERSORT algorithms were used to estimate the correlation between immune cell infiltration and the Anoscore. The ESTIMATE algorithm was employed to calculate immune scores, stromal scores and ESTIMATE scores for both low- and high-Anoscore groups. Additionally, the expression of common immune checkpoints (ICPs) was assessed between the two subgroups based on the Anoscore. ICPs with significant differential expression (P < 0.05) were visualized using boxplots.

### 2.8 Cell culture

The human EC cell line KYSE-150, the GC cell line HGC-27 and the CRC cell line Caco-2 were acquired from the China Center for Type Culture Collection (CCTCC; Wuhan, China). KYSE-150 cells and HGC-27 cells were cultured in RPMI 1640 media (Gibco, New York, USA) supplemented with 10% fetal bovine serum (FBS, Gibco, New York, USA) and 1% antibiotics (Zqxzbio, Shanghai, China), while Caco-2 cells were cultured in DMEM (Gibco, New York, USA) supplemented with 15% FBS and 1% antibiotics. All the cells were incubated at 37°C with 5% CO_2_.

### 2.9 Cell transfection

Since S100A11 and TLR3 were identified as the most significant contributors to the Anoscore, we investigated their biological functions in detail. S100A11 was knocked down with a small interfering RNA (siRNA) in KYSE-150, HGC-27 and Caco-2 cells (the sequences were listed in **Additional File 1**, **Supplementary Table S1**). The siRNAs and the negative control were transfected into cells using Lipofectamine 3000 (Invitrogen, Carlsbad, CA, USA). The pcDNA3.1-control and pcDNA3.1-TLR3 plasmids were designed and synthesized (Boshang Biotechnology, Jinan, China). The plasmids were transfected into the aforementioned cells using Lipofectamine 3000 (Invitrogen, Carlsbad, CA, USA). Quantitative real-time polymerase chain reaction (qRT-PCR) and western blotting (WB) were used to confirm the efficiency of knockdown and overexpression.

### 2.10 Cell proliferation assay

Cell proliferation was detected using the Cell Counting Kit-8 (CCK-8) (Bioss, Beijing, China). Transfected cells (3×10^3^ cells/well) and the corresponding controls were seeded into 96-well plates (Corning Incorporated, Corning, NY, USA). After culture for 24 h, 48 h, 72 h or 96 h, 10 microliters (μl) of the CCK-8 solution was added to each well, which contained 90 μl of serum-free medium. The cells were incubated for 2 h at 37 °C. Finally, we measured the absorbance with a spectrophotometer (Tecan, Männedorf, Switzerland) at 450 nm and generated growth curves.

### 2.11 Cell migration assay

Transfected cells (KYSE-150, HGC-27 and Caco-2 cells) (1×10^5^ cells/well) with 200 μl of serum-free RPMI 1640 media or DMEM were seeded into the upper chamber of a 24-well transwell system (Corning Incorporated, Corning, NY, USA) in triplicate, while 800 μl of RPMI 1640 media or DMEM containing 20% FBS was added to the lower chamber. After a certain number of hours, the migrated cells were fixed with methanol, and then a crystal violet solution (0.1%) was used to stain them. The migrated cells were observed and counted under a microscope (100×) (Olympus, Tokyo, Japan).

### 2.12 Colony formation assay

S100A11-knockdown and TLR3-overexpressing cells were plated into 6-well plates at a density of 1000 cells/well. After 10 days of incubation, the cells were fixed with 4% paraformaldehyde and stained with 0.5% crystal violet. Visible colonies (more than 50 cells) were counted.

### 2.13 qRT-PCR

Total RNA was extracted using the RNA Fast 2000 (Fastagen, Shanghai, China). The RNA concentration and purity were measured, with the quality criterion of A260/A280 ratio between 1.8 and 2.0. For cDNA synthesis, 2.0 micrograms (μg) of total RNA was reverse transcribed in a 20 μl reaction using Evo M-MLV RT Premix for qPCR (Accurate Biology, Hunan, China). The reverse transcription reaction was carried out at 37 °C for 15 minutes, followed by 85 °C for 5 seconds to inactivate the enzyme. qPCR was performed using the BlazeTaq SYBR Green qPCR mix 2.0 Kit (Accurate Biology, Hunan, China). The cycling conditions were as follows: initial denaturation at 95°C for 30 seconds, with 40 cycles consisting of denaturation at 95°C for 5 seconds and annealing and extension at 60°C for 30 seconds. All RNA extraction and qRT-PCR experiments were conducted according to the manufacturers’ instructions. The relative expression levels were calculated using the relative 2^-ΔΔCT^ method with β-actin as the internal control. The sequences of primers used were listed in **Additional File 1**, **Supplementary Table S2**.

### 2.14 WB analysis

The cells were lysed on ice with radioimmunoprecipitation assay (RIPA) lysis buffer (Solarbio, Beijing, China) containing phenylmethylsulfonyl fluoride (1 mM) and then homogenized via ultrasonication. The protein concentration was determined with Omni-Easy™ Instant BCA Protein Assay Kit (EpiZyme, Shanghai, China). The cell lysate was mixed with loading buffer (Beyotime, Shanghai, China) and denatured at 95°C for 10 minutes. The protein samples were electrophoresed on sodium dodecylsulfate−polyacrylamide gels and transferred to polyvinylidene fluoride membranes (Millipore, MA, USA). The membranes were blocked with 5% milk and then incubated with primary antibodies against S100A11 (1:1000, Proteintech, 10237-1-AP, China), TLR3 (1:1000, ABclonal, A11778, China), β-actin (1:2000, HUABIO, EM21002, China), BAX (1:20000, HUABIO, ET1702-53, China), Bcl-2 (1:2000, HUABIO, ET1702-53, China), Caspase-3 (1:2000, HUABIO, ET1602-39, China), active Caspase-3 (1:1000, HUABIO, ET1602-47, China), Caspase-7 (1:1000, HUABIO, JE59-36, China), active Caspase-7 (1:1000, HUABIO, ER60002, China), PARP1 (1:10000, Proteintech, 66520-1-Ig, China), and cleaved PARP1 (1:10000, Proteintech, 60555-1-Ig, China) overnight at 4°C. After an incubation with a secondary antibody (1:5000) for 1 hour at room temperature, exposure and visualization were conducted.

### 2.15 Statistical analysis

Statistical analysis was conducted using R (version 4.2.2), GraphPad Prism (version 8.0) and SPSS (version 23.0) software. The Wilcoxon test was used for the comparison of two independent samples. T-tests and one-way ANOVA were used to compare parametric data. The Kruskal-Wallis test was used to compare nonparametric data across multiple samples. A p-value < 0.05 was considered statistically significant.

## 3 Results

### 3.1 Expression of ARGs and genetic variations in GI cancers

**Figure 1:**
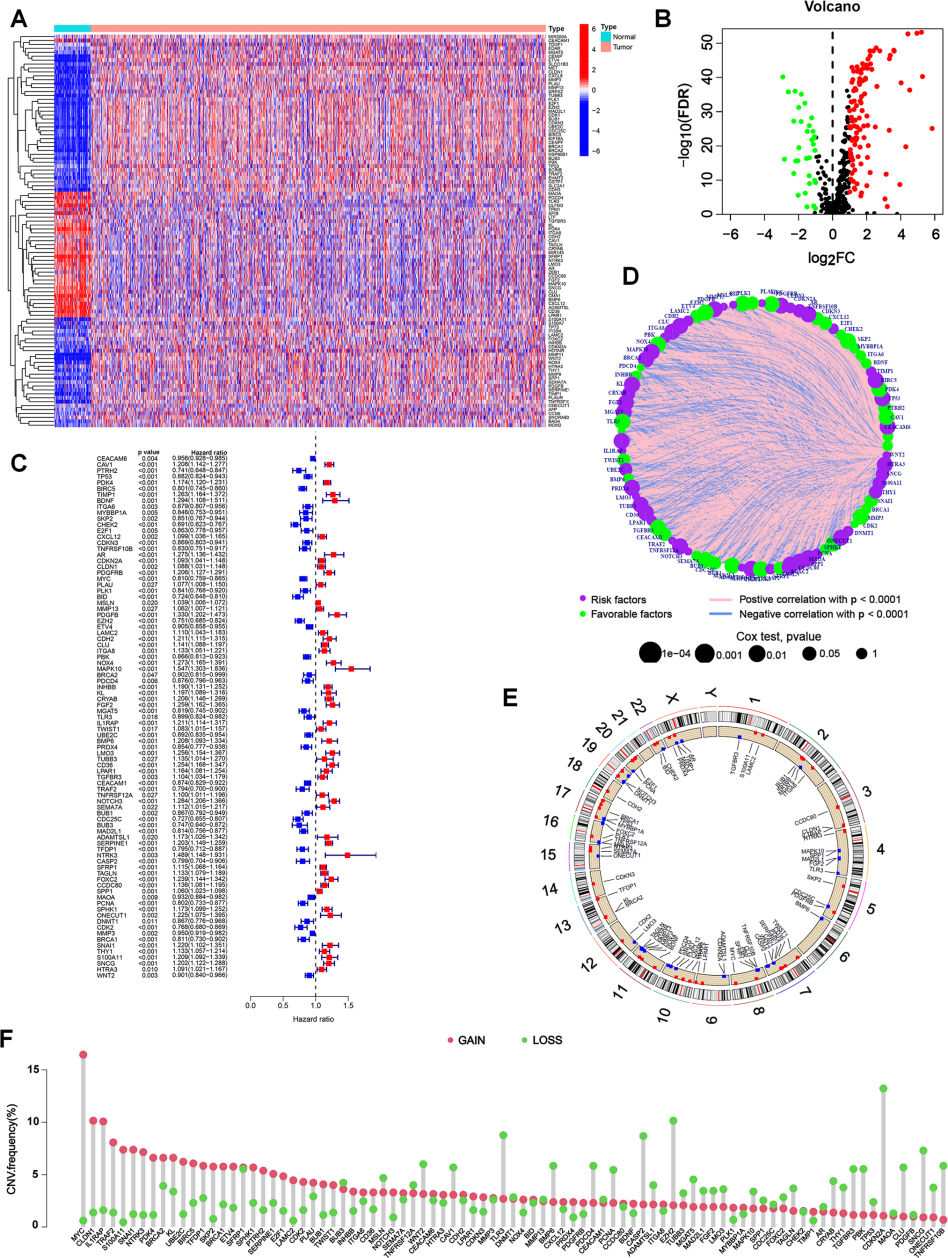
Expression of ARGs and genetic variations in GI cancers. **(A)** Heatmap of 142 differentially expressed ARGs between tumor and normal tissues from TCGA database. **(B)** Volcano plot showing differentially expressed ARGs with an FDR < 0.05 and |log2FC| >1. Genes upregulated in tumors were marked in red, downregulated genes in blue, and non-significant genes in black. **(C)** Univariate Cox regression analysis to identify prognostic ARGs (p < 0.05). Hazard ratios and confidence intervals were provided for each ARG. **(D)** Network plot showing the correlation of ARGs in GI cancers. The size of each circle represented the p values of ARGs in the prognostic analysis, while the lines represented the interaction strengths between ARGs. **(E)** Copy number variations (CNVs) in ARGs are visualized on chromosomes. **(F)** Bar chart visualizing the CNV frequencies of 87 prognostic ARGs in GI cancers.

We obtained RNA transcriptome data and corresponding clinical data from ESCA (N = 185), STAD (N = 443), COAD (N = 459) and READ (N = 168) patients in TCGA database. Among the 647 ARGs extracted from the GeneCards and Harmonizome databases, 142 differentially expressed ARGs were recognized between tumor and normal tissues, including 109 upregulated and 33 downregulated genes in tumors (**Figures 1A, B**). After combining the expression data and clinical information of TCGA, GSE39582 and GSE84437 cohorts, we performed a univariate Cox analysis to identify differentially expressed ARGs associated with the prognosis. The results suggested that 87 ARGs were associated with the survival status and survival events of patients. Among them, the expression of 49 genes, such as CHEK2, BID, CDC25C and PTRH2, was negatively correlated, and the expression of 38 genes, such as MAPK10, NTRK3, PDGFB and BDNF, was positively correlated with survival (**Figure 1C**). A network plot was constructed to visualize the intricate relationship between ARGs and their prognostic value for GI tumors (**Figure 1D**). Copy number variations (CNVs) in ARGs were visualized on chromosomes (**Figure 1E**). Our investigation of 87 ARGs revealed that CNV-related mutations were prevalent, with 49 genes exhibiting widespread CNV amplification and 38 genes exhibiting CNV deletions (**Figure 1F**).

### 3.2 Stratification of patients based on ARG expression patterns

**Figure 2:**
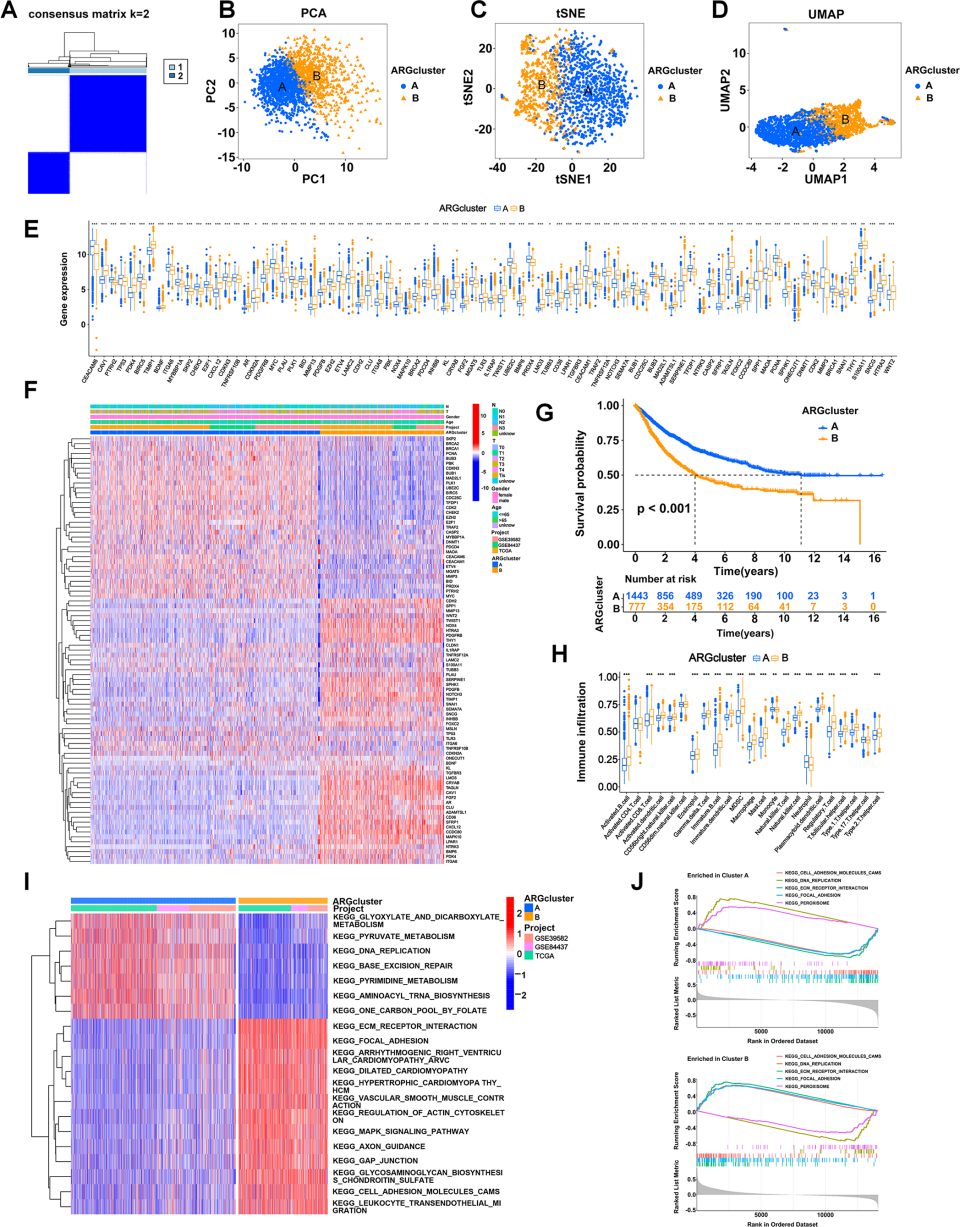
Stratification of patients based on ARG expression patterns. **(A)** Two anoikis patterns were established via unsupervised clustering analysis. **(B-D)** Visualization of the clustering results using three dimensionality reduction methods: PCA **(B)**, t-SNE **(C)**, and UMAP **(D)**, confirming the separation of ARG clusters. **(E)** Boxplot showing differentially expressed ARGs between ARG clusters A and B. **(F)** Heatmap visualizing the detailed expression of ARGs and clinical characteristics of clusters A and B. Rows represented genes, and columns represented patients, with hierarchical clustering demonstrating clear separation of subgroups. **(G)** KM analysis comparing the OS of patients in ARG clusters A and B. **(H)** Abundances of immune cells in the two clusters. **(I)** Heatmap showing the pathways activated in ARG clusters analyzed using GSVA. **(J)** The pathways enriched in ARG clusters A and B were analyzed using GSEA. *p < 0.05;**p < 0.01; and ***p < 0.001.

We performed an unsupervised clustering analysis to examine whether anoikis-related patterns could be used to classify patients with GI cancers based on the expression of the 87 prognostic ARGs. The results showed that k = 2 was the optimal parameter, which meant that patients were classified into two anoikis-related patterns, termed ARG clusters A and B (**Figure 2A**). ARG clusters A and B were distinguished dramatically via PCA (**Figure 2B**), t-SNE (**Figure 2C**), and UMAP (**Figure 2D**) analyses, which validated our anoikis-related patterns. A boxplot was generated to visualize the differentially expressed ARGs in the two clusters, in which 37 ARGs, such as CHEK2, CDC25C and TRAF2, were upregulated in ARG cluster A, and 48 ARGs, such as PDK4, CXCL12, MAPK10 and NOTCH3, were upregulated in ARG cluster B (**Figure 2E**). A heatmap was generated to visualize the detailed expression of ARGs and the clinical characteristics of clusters A and B (**Figure 2F**). The KM analysis revealed a difference in patient survival between ARG clusters A and B. The prognosis of patients in ARG cluster B was dramatically worse than that of patients in ARG cluster A (p < 0.001) (**Figure 2G**). By analyzing immune cell infiltration via ssGSEA, we observed that immune cells, such as CD8-positive T lymphocytes (CD8^+^ T cells), activated natural killer (NK) cells, dendritic cells (DCs), macrophages, and activated B cells, were more abundant in ARG cluster B (**Figure 2H**). These results indicated that anoikis-related patterns were correlated with the immune landscape of GI cancers. ARG cluster A tended to represent cold tumors, whereas ARG cluster B tended to represent hot tumors. Thus, patients in ARG cluster B might be more sensitive to immunotherapy. GSVA revealed that multiple cancer-related pathways, such as ECM-receptor interaction, focal adhesion, regulation of the actin cytoskeleton, the mitogen-activated protein kinase (MAPK) signaling pathway, gap junctions and cell adhesion molecules, were significantly activated in ARG cluster B (**Figure 2I**). GSEA revealed that DNA replication and peroxisomes were more strongly activated in ARG cluster A (**Figure 2J**). In addition, ECM-receptor interaction, focal adhesion and cell adhesion molecules were more enriched in ARG cluster B, which was consistent with the results of GSVA (**Figure 2J**).

### 3.3 Construction and validation of the Anoscore

**Figure 3:**
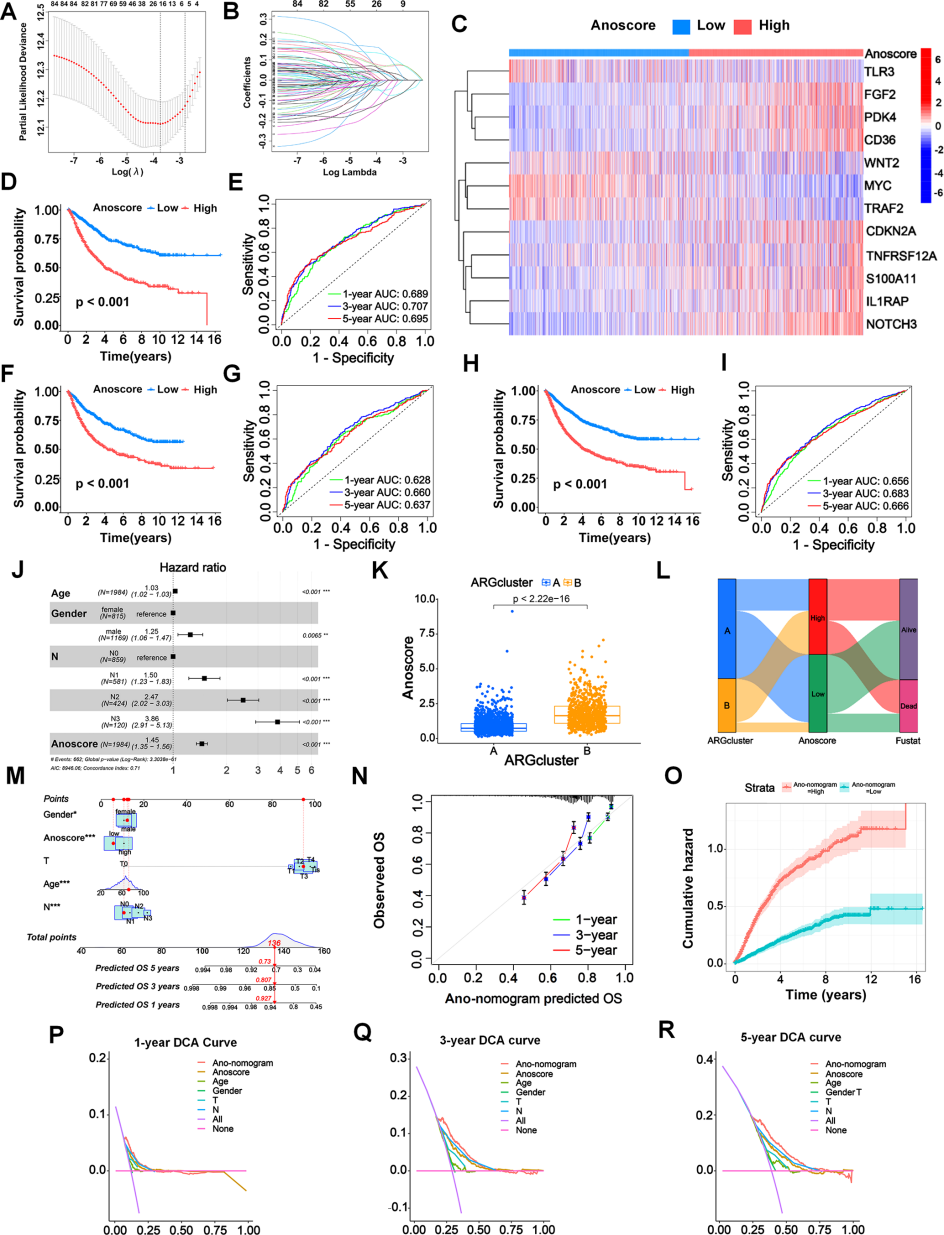
Construction and validation of an anoikis-related prognostic signature (Anoscore) and nomogram (Ano-nomogram). **(A, B)** LASSO algorithm **(A)** and multivariate Cox regression analysis **(B)** identified 12 prognostic ARGs from TCGA and GEO datasets. **(C)** A heatmap was generated to visualize the expression of the 12 candidate genes that comprised Anoscore in the low- and high-Anoscore groups. **(D)** KM survival analysis for the training cohort (n=1110), showing that patients in the high-Anoscore group had significantly worse OS than those in the low-Anoscore group. **(E)** ROC curves of the Anoscore in the training cohort (n=1110), demonstrating the predictive accuracy of the Anoscore for OS. **(F, G)** External validation of the Anoscore in the test cohort (n=1110), with KM survival analysis **(F)** confirming the significant prognostic difference and ROC curve analysis **(G)** verifying prediction consistency. **(H, I)** A comprehensive analysis of the full patient cohort (n=2220) revealed consistent trends in the KM survival curve **(H)**, while the ROC curve **(I)** further validated the prognostic capability of Anoscore. **(J)** Multivariate Cox regression analysis of the relationships among clinicopathological factors, the Anoscore and survival. **(K)** The correlation analysis between the Anoscore and ARG clusters, highlighting the integration of molecular subtypes and prognostic scores. **(L)** Sankey diagram showing the interrelationships among ARG clusters, the Anoscore and the survival status of patients, providing a comprehensive view of patient stratification. **(M)** Ano-nomogram integrating the Anoscore with clinical features to predict 1-, 3-, and 5-year OS probabilities for individual patients. **(N)** The calibration curve illustrated the actual and predicted survival probabilities using the Ano-nomogram, demonstrating good alignment. **(O)** Nelson−Aalen cumulative risk curves were used to visualize the cumulative hazards of different Ano-nomogram groups. **(P-R)** Decision curve analyses at 1 **(P)**, 3 **(Q)**, and 5 **(R)** years for the Ano-nomogram, Anoscore and clinical factors. *p < 0.05; **p < 0.01; and ***p < 0.001.

We systematically divided patients into a training cohort (n =1110) and a test cohort (n = 1110). Then we used the LASSO algorithm (**Figure 3A**) and multivariate Cox analysis (**Figure 3B**) to further identify the significant prognostic ARGs for patients in the training cohort. Finally, we obtained 12 ARGs with p < 0.05 that were utilized to construct the prognostic signature. The risk score of each patient was calculated as follows: Anoscore = 0.179 × ExpPDK4 + 0.078 × ExpCDKN2A − 0.180 × ExpMYC + 0.130 × ExpFGF2 – 0.291 × ExpTLR3 + 0.124 × ExpIL1RAP + 0.120 × ExpCD36 − 0.222 × ExpTRAF2 + 0.145 × ExpTNFRSF12A + 0.134 × ExpNOTCH3 + 0.260 × ExpS100A11 − 0.109 × ExpWNT2 (**Additional File 1**, **Supplementary Table S3**). The heatmap depicted the expression levels of the ARGs that comprised the Anoscore in different risk groups (**Figure 3C**). FGF2, PDK4, CD36, CDKN2A, S100A11, IL1RAP and NOTCH3 were significantly upregulated in patients with high Anoscores (**Figure 3C**).

The KM analysis revealed a significant relationship between the Anoscore and the prognosis of patients with GI cancers. ROC curves were constructed to evaluate the sensitivity and specificity of the risk model. The outcomes were assessed based on the area under the ROC curve (AUC). The model demonstrated robust performance in both cohorts. In the training cohort, significant prognostic stratification was observed, with the high-Anoscore group showing markedly shorter survival times compared to the low-Anoscore group (p < 0.001, **Figure 3D**). Furthermore, the model exhibited strong discriminative power, achieving AUCs of 0.689, 0.707, and 0.695 for 1-, 3-, and 5-year survival predictions, respectively (**Figure 3E**). Validation in the test cohort confirmed the model’s reliability, with significant prognostic stratification maintained (p < 0.001) and the low-Anoscore group consistently demonstrating better clinical outcomes (**Figure 3F**). The discriminative performance of Anoscore remained stable, with AUCs of 0.628, 0.660, and 0.637 for 1-, 3-, and 5-year survival predictions (**Figure 3G**). The similar performance metrics between the training and test cohorts indicated good model generalizability.

The KM analysis further revealed that all patients with high Anoscores had generally worse prognoses (p < 0.001) (**Figure 3H**), with 1-, 3-, and 5-year AUCs of 0.656, 0.683, and 0.666, respectively (**Figure 3I**). The robustness of Anoscore was further confirmed through cancer-type specific analyses, showing consistent performance. We evaluated the relationships between OS and the Anoscore in patients with ESCA, STAD, COAD and READ using KM analyses. Patients with high Anoscores had shorter OS times, consistent with the analysis of all patients with GI cancers (**Additional File 2**, **Supplementary Figure S1**). Similarly, the Anoscore proved highly reliable in patients with STAD, COAD and READ (**Additional File 2**, **Supplementary Figure S1**). However, perhaps due to the small sample size in the ESCA cohort, the survival analysis was not statistically significant. Moreover, the multivariate Cox analysis confirmed the Anoscore as an independent prognostic factor (hazard ratio [HR] 1.45, 95% confidence interval [CI] 1.35–1.56) (**Figure 3J**). Considering these results, a high Anoscore can be recognized as an effective indicator of unfavorable OS among patients with GI cancers.

Consistent with our previous findings, we observed the biological consistency between Anoscore and the ARG clusters (**Figure 3K**). We also illustrated the interrelationship among the two ARG clusters, Anoscore typing, and the survival status of patients in a Sankey diagram. Patients in ARG cluster A predominantly corresponded to the low-Anoscore group, of which surviving patients constituted the majority (**Figure 3L**).

### 3.4 Construction and validation of the Ano-nomogram

We constructed a novel nomogram capable of predicting survival probabilities at 1, 3, and 5 years that comprised the clinical factors and the Anoscore (**Figure 3M**). The hybrid nomogram was stable and accurate, potentially offering valuable clinical utility in patient management. Strong agreement between the actual survival probabilities and the Ano-nomogram-predicted survival probabilities was exhibited in the calibration curve (**Figure 3N**). The cumulative hazards of different groups were calculated by analyzing the Nelson-Aalen cumulative risk curve. The cumulative risk of the low Ano-nomogram group increased at a slower rate over time, indicating that patients with low Ano-nomogram scores might have better survival outcomes, as shown in **Figure 3O**. The 1-year DCA curve revealed that most of the Ano-monogram curve was in the area above the other lines, which demonstrated that using the Ano-nomogram to reach a decision on the prognosis was more reliable and could result in a greater net benefit (**Figure 3P**). The 3-year DCA curve (**Figure 3Q**) and 5-year DCA curve (**Figure 3R**) also revealed that the Ano-nomogram had outstanding performance.

### 3.5 Single-cell analysis of S100A11 and TLR3 in GI cancers

**Figure 4:**
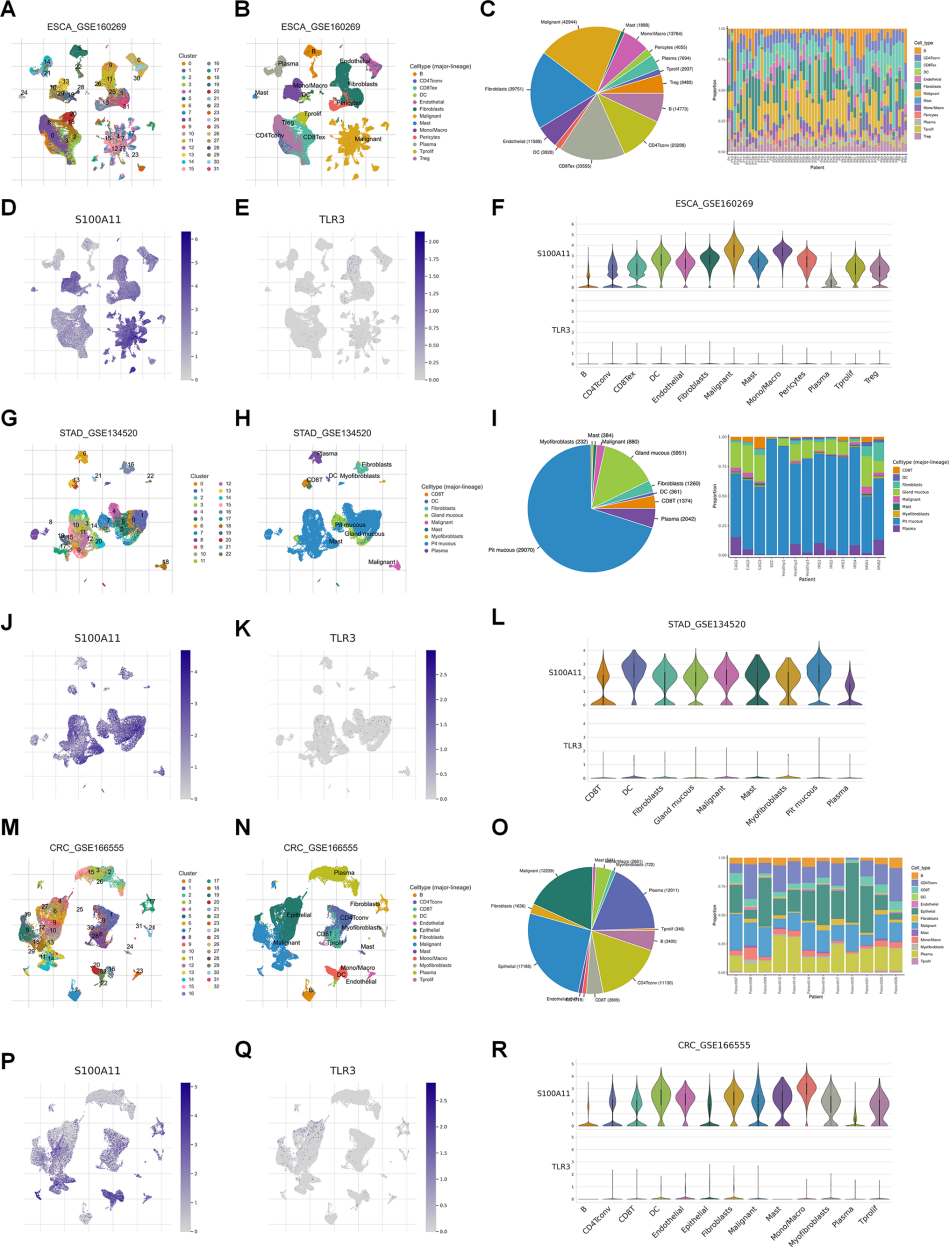
Single-cell analysis of S100A11 and TLR3 expression in GI cancers. **(A, B)** The ESCA cohort (GSE160269) was analyzed using single-cell RNA sequencing data. The number of clusters **(A)** and clustering results **(B)** were visualized, identifying distinct cell populations. **(C)** The numbers of different types of cells and the proportions of different cells in each patient in GSE160269. **(D, E)** Map showing the distribution of S100A11 **(D)** and TLR3 **(E)** across cell clusters in the ESCA cohort. **(F)** Violin plot showing the expression levels of S100A11 and TLR3 in each type of cell in GSE160269. **(G, H)** The number of clusters **(G)** and clustering results **(H)** for the STAD cohort (GSE134520). **(I)** The numbers of different types of cells and the proportions of different cells in each patient in GSE134520. **(J, K)** Map showing the distribution of S100A11 **(J)** and TLR3 **(K)** in GSE134520. **(L)** Violin plot showing the expression levels of S100A11 and TLR3 in each type of cell in GSE134520. **(M, N)** The number of clusters **(M)** and clustering results **(N)** for the CRC cohort (GSE166555). **(O)** The numbers of different types of cells and the proportions of different cells in each patient in GSE166555. **(P, Q)** Map showing the distribution of S100A11 **(P)** and TLR3 **(Q)** in GSE166555. **(R)** Violin plot showing the expression levels of S100A11 and TLR3 in each type of cell in GSE166555.

S100A11 is a calcium-binding protein that regulates cellular processes including cell cycle and differentiation (8), while TLR3 is a pattern recognition receptor crucial for immune response and cell death signaling (9). According to our previous LASSO algorithm and multivariate Cox analyses, S100A11 had the largest positive coefficient, and TLR3 had the largest negative coefficient. Therefore, we investigated the expression of S100A11 and TLR3 in single-cell sequencing data from TISCH2. In EC patients (GSE160269), the expression of these two genes could be clustered into 31 groups (**Figure 4A**), and the clustering scatter plot showed the clustering results (**Figure 4B**). The pie chart showed the number of different cell types, and the stacked bar chart showed the proportions of different cells in each patient (**Figure 4C**). S100A11 was highly distributed and expressed in malignant cells, while TLR3 expression was lower (**Figures 4D, E**). The distribution and expression of S100A11 and TLR3 in different cell types of the dataset were clearly revealed in the violin plot (**Figure 4F**). The results revealed that S100A11 was expressed mainly in tumor cells, monocytes/macrophages and fibroblasts in GSE160269 (**Figure 4F**). In contrast, TLR3 was expressed at very low levels in all cell types, especially in tumor cells (**Figure 4F**). In GSE134520 (GC), the clustering analysis revealed 22 clusters (**Figures 4G, H**), of which pit mucous cells were the majority (**Figure 4I**). The distribution and expression of S100A11 and TLR3 expression in GSE134520 were shown in the map (**Figures 4J, K**). S100A11 was enriched in mast cells, DCs, malignant cells and myofibroblasts (**Figure 4L**). Meanwhile, TLR3 was enriched in DCs and myofibroblasts (**Figure 4L**). With respect to CRC patients, the clustering analysis clustered the GSE166555 dataset into 32 groups (**Figure 4M**). The distribution of each cell type was shown in **Figure 4N**. The proportion of each cell type was shown in **Figure 4O**. S100A11 was expressed at high levels in monocytes/macrocytes, DCs and malignant cells, whereas TLR3 was expressed at slightly higher levels in fibroblasts and endothelial cells and at low levels in other cells (**Figures 4P–R**).

### 3.6 Immune landscape of the low- and high- Anoscore subgroups

**Figure 5:**
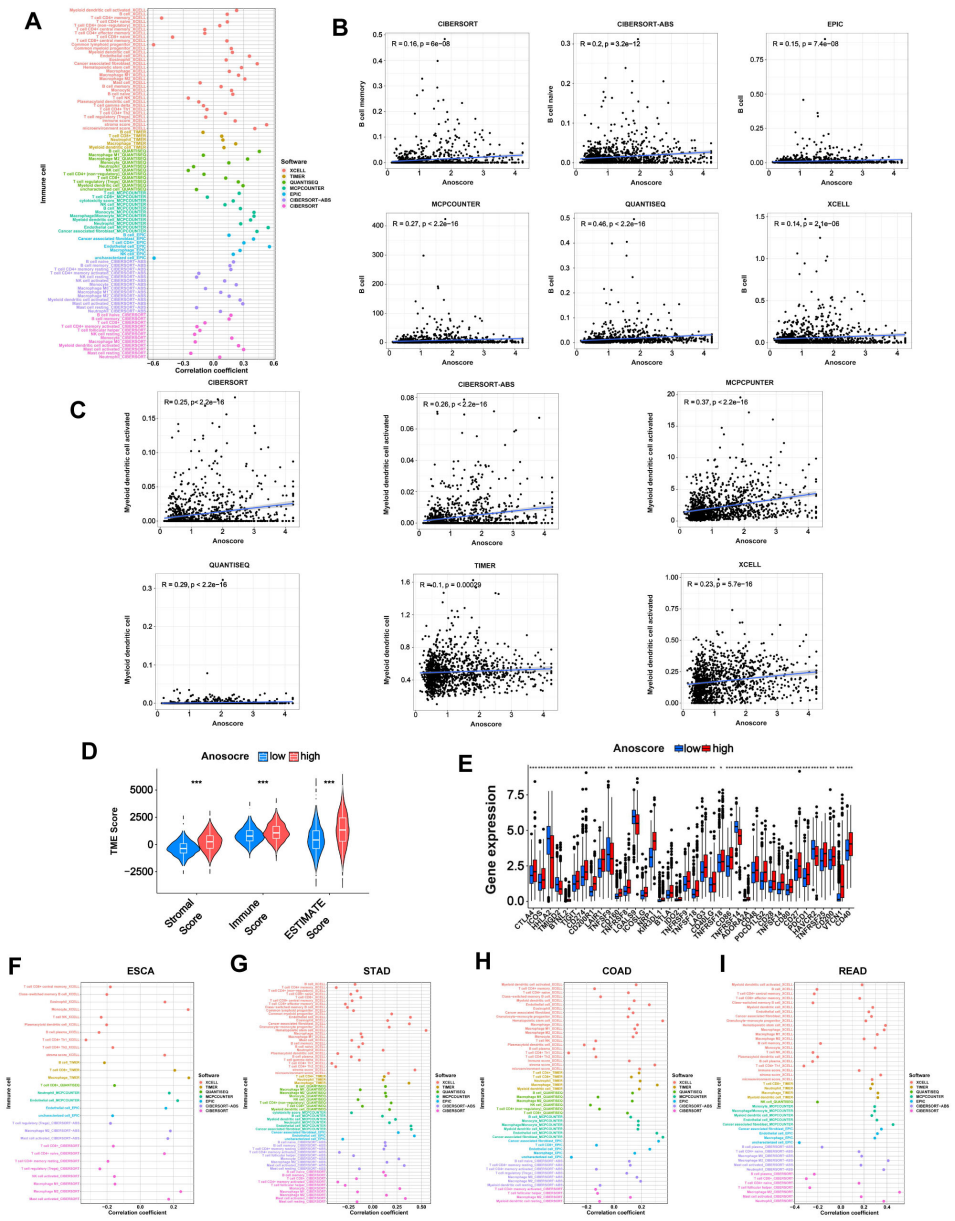
Immune landscape of the low- and high-Anoscore groups. **(A)** Bubble chart showing the relationship between the Anoscore and immune cell infiltration in GI cancers. **(B)** The correlation between the Anoscore and B cells in CIBERSORT, CIBERSORT-ABS, EPIC, MCPCOUNTER, QUANTISEQ, and XCELL, showing consistent trends across methods. **(C)** The correlation between the Anoscore and DCs in CIBERSORT, CIBERSORT-ABS, MCPCOUNTER, QUANTISEQ, TIMER and XCELL. **(D)** Differences in stromal scores, immune scores and ESTIMATE scores between the low- and high-Anoscore groups. **(E)** Differences in the expression of immune checkpoints, such as PD-1, CTLA-4, and others, between the low- and high-Anoscore groups. **(F-I)** Detailed correlations between the Anoscore and the infiltration of immune cells in ESCA **(F)**, STAD **(G)**, COAD **(H)** and READ **(I)**.

Immunotherapy plays an important role in tumor treatment. Considering the relatively low response rate to immunotherapy and the lack of promising predictors, we conducted an immune-related analysis to evaluate whether the Anoscore was associated with the immune status and could predict the immunotherapy response in GI tumors. We used seven different software packages to estimate the correlation between the Anoscore and tumor-infiltrating immune cells. We found that the high Anoscore was significantly related to increased immune cell infiltration, as shown in the immune cell bubble chart (**Figure 5A**). In general, B cells were positively correlated with the Anoscore across the above cancer types according to CIBERSORT, CIBERSORT-ABS, EPIC, MCPCOUNTER, QUANTISEQ, and XCELL (**Figure 5B**). DCs were positively correlated with the Anoscore in CIBERSORT, CIBERSORT-ABS, MCPCOUNTER, QUANTISEQ, TIMER and XCELL (**Figure 5C**). Macrophages and neutrophils were positively correlated with the Anoscore in 6 and 4 datasets, respectively (CIBERSORT-ABS, EPIC, MCPCOUNTER, QUANTISEQ, TIMER, and XCELL for macrophages and CIBERSORT, CIBERSORT-ABS, MCPCOUNTER, and TIMER for neutrophils; **Additional File 2**, **Supplementary Figures S2A, B**). In addition, with CIBERSORT-ABS, EPIC and XCELL, the number of CD4-positive T lymphocytes (CD4^+^ T cells) was positively correlated with the Anoscore (**Additional File 2**, **Supplementary Figure S2C**). Moreover, CD8^+^ T cells were positively correlated with the Anoscore according to the QUANTISEQ, TIMER and XCELL tools (**Additional File 2**, **Supplementary Figure S2D**). NK cells (**Additional File 2**, **Supplementary Figure S2E**) and monocytes (**Additional File 2**, **Supplementary Figure S2F**) were also positively correlated with the Anoscore. Overall, tumors in the high-Anoscore group exhibited much greater immune cell infiltration, indicating that these tumors were hot and could respond to immunotherapy more sensitively. According to TME scores, the infiltration of stromal cells and immune cells was greater in the high-Anoscore group than in the low-Anoscore group, indicating that the TME differed between the low- and high-Anoscore groups (**Figure 5D**). We also analyzed the relationships between the Anoscore and ICPs. Cooccurrences of ligands and receptor expression were identified. Nine pairs, including PDCD1LG2/CD274-PDCD1, CD86-CD28/CTLA4, TNFSF18-TNFRSF18, ICOSLG-ICOS, TNFRSF14-CD160/TNFSF14/BTLA, TNFSF9-TNFRSF9, CD40-CD40LG, LGALS9-HAVCR2 and CD200-CD200R1, were highly expressed in the high-Anoscore group (**Figure 5E**), suggesting that appropriate immune checkpoint inhibitors could be effectively applied, based on the Anoscore for GI cancers. Next, we evaluated the associations between the Anoscore and the infiltration of immune cells in ESCA (**Figure 5F**), STAD (**Figure 5G**), COAD (**Figure 5H**) and READ (**Figure 5I**). Among these four tumors, the high Anoscore was associated with increased immune cell infiltration, which is consistent with the previous overall data.

### 3.7 Correlation of the Anoscore with drug sensitivity

**Figure 6:**
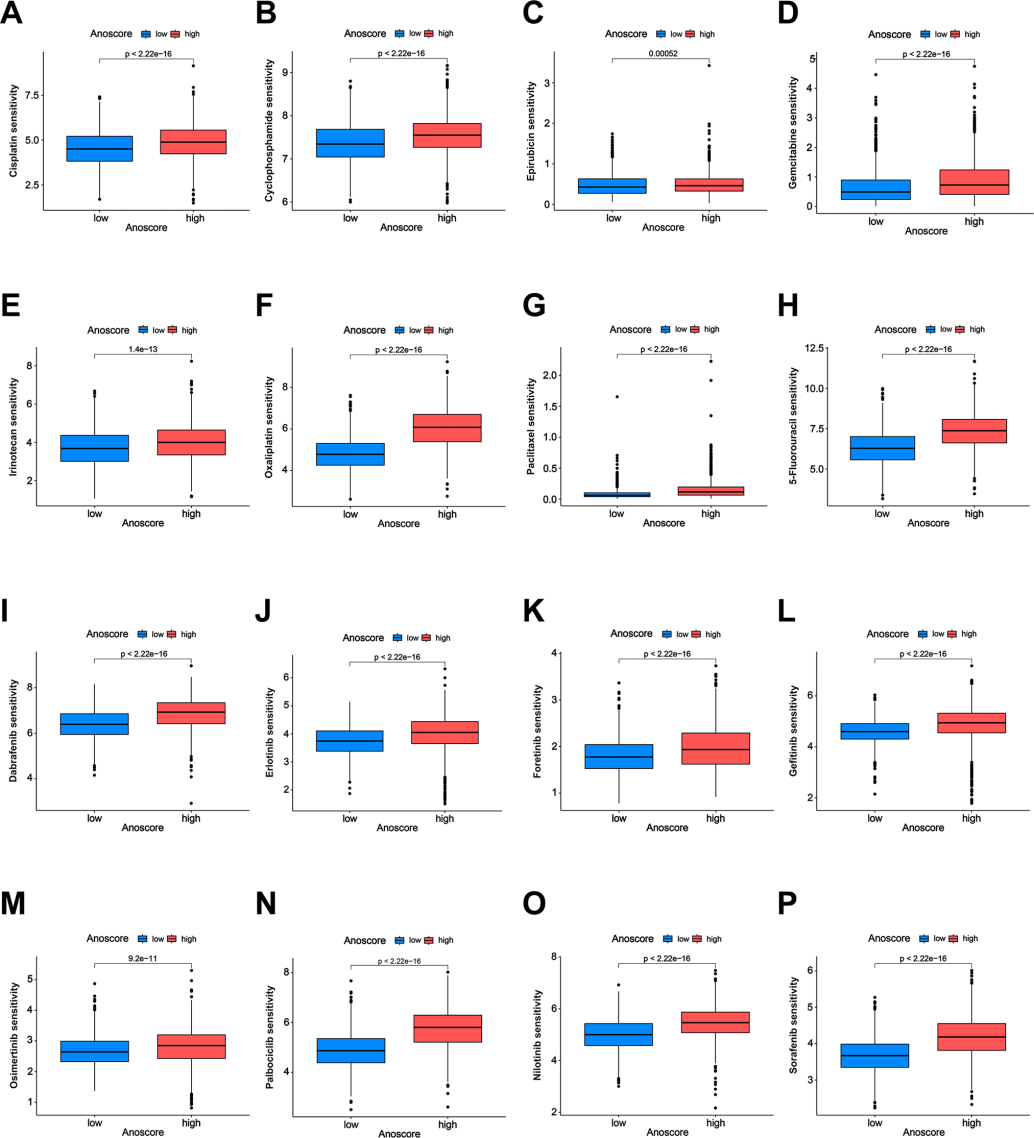
Correlation of the Anoscore with drug sensitivity. **(A-H)** Analysis of the sensitivity of the high- and low-Anoscore groups to chemotherapy drugs, including cisplatin **(A)**, cyclophosphamide **(B)**, epirubicin **(C)**, gemcitabine **(D)**, irinotecan **(E)**, oxaliplatin **(F)**, paclitaxel **(G)** and 5-fluorouracil **(H)**. **(I–P)** Analysis of the sensitivity of the high- and low-Anoscore groups to targeted drugs, including dabrafenib **(I)**, erlotinib **(J)**, foretinib **(K)**, gefitinib **(L)**, osimertinib **(M)**, palbociclib **(N)**, nilotinib **(O)** and sorafenib **(P)**.

Drug treatment, including chemotherapy and targeted drugs, plays a crucial role in cancer management. However, the therapeutic outcomes vary significantly due to differences in drug sensitivity. We evaluated the value of the Anoscore in predicting drug sensitivity in GI cancers. The low- and high-Anoscore groups were differentially sensitive to 165 types of drugs in the GDSC2 database. Among them, the high-Anoscore group was more sensitive to 141 types of drugs, whereas the low-Anoscore group was more sensitive to 24 types of drugs (**Additional File 1**, **Supplementary Table S4**). The high-Anoscore group was more sensitive to multiple chemotherapy drugs, including cisplatin, paclitaxel, oxaliplatin, irinotecan, gemcitabine, cyclophosphamide, epirubicin and 5-fluorouracil (**Figures 6A–H**, **Additional File 2**, **Supplementary Figure S3**). Similar results were obtained with targeted medicine. The patients in the high-Anoscore group exhibited increased sensitivity to various targeted medicines, including dabrafenib, erlotinib, foretinib, gefitinib, nilotinib, sorafenib, osimertinib and palbociclib (**Figures 6I–P**, **Additional File 2**, **Supplementary Figure S3**). The low-Anoscore group was more sensitive to drugs that included dasatinib, doramapimod, IGF1R_3801, AMG-319, AZD1332, AZD2014 and AZD5582 (**Additional File 2**, **Supplementary Figure S3**, **Additional File 1**, **Supplementary Table S4**). Our analysis suggested that the two groups had different sensitivities to medicine. Overall, patients with high Anoscores were more sensitive to drug treatment, especially chemotherapy and targeted therapy.

### 3.8 *In vitro* validation of S00A11 and TLR3

**Figure 7:**
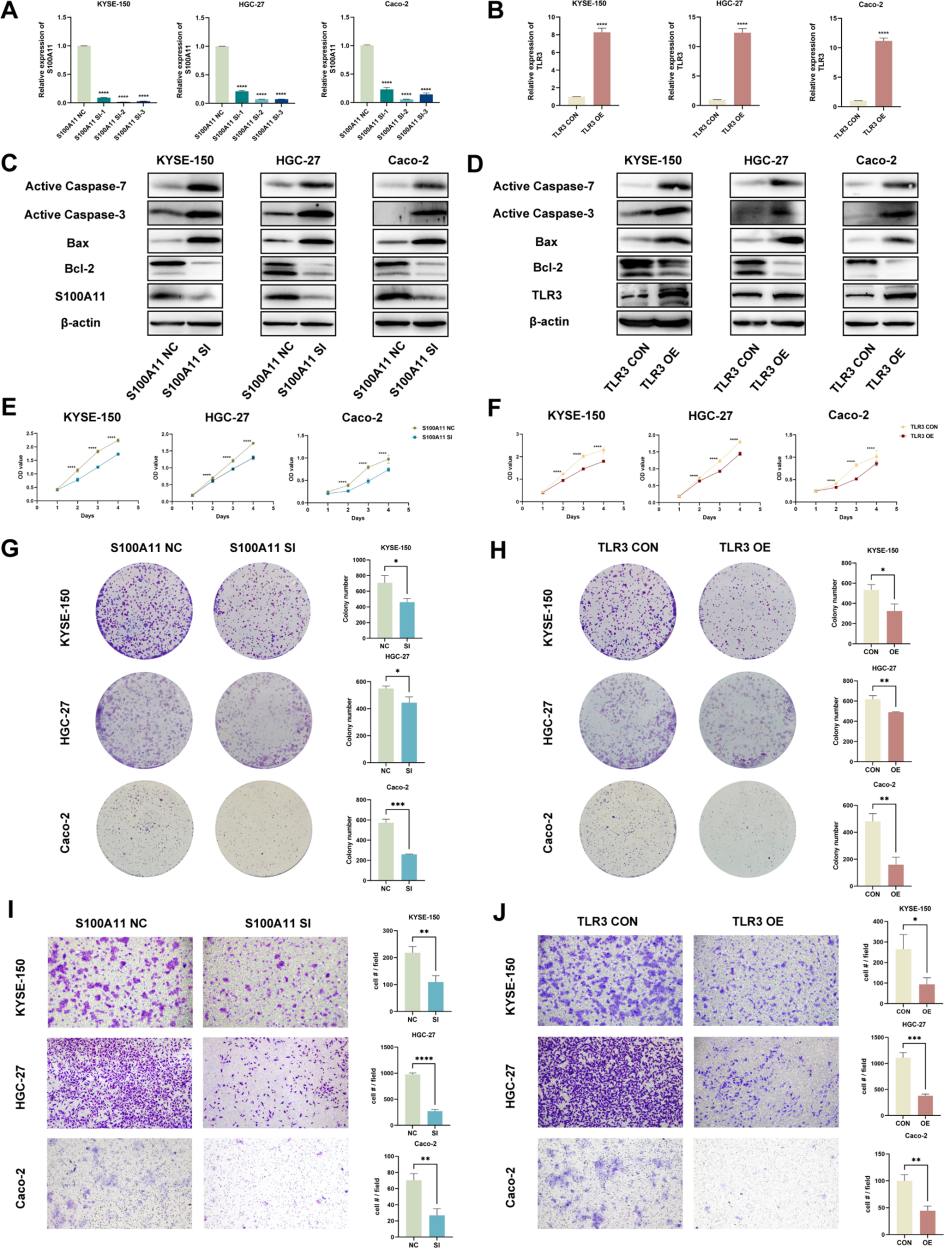
*In vitro* validation of candidate genes (S100A11 and TLR3). **(A, B)** The validation of S100A11 downregulation **(A)** and TLR3 upregulation **(B)** by qRT-PCR. **(C, D)** Validation of the downregulation of the S100A11 protein and upregulation of the TLR3 protein, as well as the expression of anoikis indicators, in S100A11-depleted **(C)** and TLR3-overexpressing **(D)** cells by WB. **(E, F)** Cell proliferation assays for S100A11-depleted **(E)** and TLR3-overexpressing **(F)** KYSE-150, HGC-27 and Caco-2 cells using CCK-8 method. **(G, H)** Colony formation assays of S100A11-depleted **(G)** and TLR3-overexpressing **(H)** KYSE-150, HGC-27 and Caco-2 cells. **(I, J)** Transwell migration assays of S100A11-depleted **(I)** and TLR3-overexpressing **(J)** KYSE-150, HGC-27 and Caco-2 cells. *p < 0.05; **p < 0.01; ***p < 0.001; and ****p < 0.0001.

We conducted experiments to explore the biological functions of S100A11 and TLR3 *in vitro*. KYSE-150, HGC-27 and Caco-2 cells with S100A11 knockdown and TLR3 overexpression were constructed (**Figures 7A, B**). Afterward, we evaluated the expression of apoptosis-related proteins via WB analysis. Ultralow-attachment 6-well plates were used to suspend EC, GC and CRC cells, analogous to the conditions of anoikis. After cells were cultured in suspension for 24 h, S100A11 knockdown and TLR3 overexpression increased the expression of active caspase 3, active caspase 7, cleaved PARP and Bax in KYSE-150, HGC-27 and Caco-2 cells but reduced the expression of Bcl-2 (**Figures 7C, D**, **Additional File 2**, **Supplementary Figure S4**). CCK-8 assays revealed that S100A11 knockdown and TLR3 overexpression inhibited cell proliferation (**Figures 7E, F**). Furthermore, we conducted colony formation assays, which revealed fewer colonies in the S100A11-knockdown and TLR3-overexpressing groups than in the negative control groups (**Figures 7G, H**). The results of transwell migration experiments revealed that S100A11 knockdown and TLR3 overexpression decelerated the migration of cells (**Figures 7I, J**). These results indicated that S100A11 might promote the proliferation and migration of tumor cells, whereas TLR3 might have the opposite effects.

In summary, S100A11 knockdown and TLR3 overexpression promoted anoikis and diminished the malignancy of tumor cells, indicating that S100A11 expression might be positively correlated with anoikis resistance and a poor prognosis, whereas TLR3 expression was negatively correlated. The *in vitro* validation of S100A11 and TLR3 functions was consistent with the results of our bioinformatics analysis described above, enhancing the reliability of our research.

## 4 Discussion

GI cancers, including EC, GC, CC and RC, have a high burden and pose a serious threat to the lives of the general public. They are characterized by easy metastasis, invasion, and a poor prognosis (10). However, effective predictive biomarkers are still lacking. Anoikis resistance has been proven to play a crucial role in tumor metastasis and growth. After tumor cells are isolated from the primary site, they need to obtain anoikis resistance for survival and expansion. Nevertheless, the impact of ARGs on the prognosis of patients with GI cancers still needs further exploration.

In our study, we used an unsupervised clustering algorithm and ultimately classified patients with GI cancers into two anoikis-related patterns. Our analysis revealed clear differences in the survival time of patients between the two subgroups. Patients in ARG cluster B exhibited poorer prognosis compared to those in ARG cluster A. Tumors in cluster B might possess a higher degree of malignancy, which could contribute to increased metastasis and reduced survival time. Furthermore, we studied immune cell infiltration between clusters A and B, and notable differences were found. These findings indicate that anoikis is related to the immune microenvironment, which strongly influences tumorigenesis. In summary, our findings demonstrated that ARGs influence the prognosis and immune responses of patients with GI cancers.

Research on the development of reliable prognostic biomarkers for GI cancers is currently a focal point. We constructed the Anoscore, a risk model based on ARGs, using univariate, LASSO and multivariate Cox regression analyses. The Anoscore is the first anoikis-related prognostic model that covers four types of GI cancers. In this analysis, the twelve most significant ARGs were obtained. Next, we verified the accuracy of the Anoscore in predicting the prognosis of patients with GI cancers, and the results revealed that the Anoscore had great predictive ability. Moreover, our analysis revealed that the Anoscore was an independent prognostic factor; thus, we combined the Anoscore with clinical factors to construct the Ano-nomogram, which was capable of forecasting survival probabilities with good performance. The Anoscore and Ano-nomogram are useful tools in clinical practice for predicting the prognosis of patients with GI cancers.

Among the 12 candidate genes, S100A11, TNFRSF12A, NOTCH3, FGF2, IL1RAP, CD36 and CDKN2A were positively correlated with the Anoscore, whereas TLR3, TRAF2, MYC, and WNT2 were negatively correlated. Most genes associated with the Anoscore were relevant to the prognosis and progression of GI cancers. S100A11 and TLR3 contributed the most to the Anoscore. The main function of S100A11, also known as S100C, is to transduce calcium-dependent cell signals and regulate the cell cycle, cell differentiation and extracellular matrix secretion. New evidence suggests that S100A11 is highly expressed in many cancers (11–13), and is closely related to malignant proliferation, distant metastasis and a poor prognosis (14–16). Toll-like receptor 3 (TLR3) is a pattern recognition receptor that plays a critical role in the immune response. Most studies have focused on the beneficial role of TLR3 in tumor cells, which can lead to the production of cytotoxic cytokines and interferons that promote caspase-dependent cell apoptosis (17). Research has indicated that high expression of TLR3 is correlated with a decreased risk of some tumors and favorable clinical outcomes (18–21). In addition, TLR3 agonists can activate tumor-specific immune responses in mice and patients (22–25). These findings concerning the biology of S100A11 and TLR3 aligned with our findings. We comprehensively verified the expression and biological behaviors of S100A11 and TLR3 via multiple approaches, including single-cell analyses, qRT-PCR, WB and functional assays. Their biological functions were consistent with previous studies. The Anoscore and these candidate genes may become new potential targets and biomarkers for GI cancers. However, further studies of these genes are needed to explore the specific mechanisms involved.

Immunotherapy has been successfully applied in clinical practice and has been proven to improve the survival of patients with EC, GC and CRC (26–30). Nevertheless, effective biomarkers for predicting the response of GI cancers to immunotherapy remain elusive. We explored the relationship between the Anoscore and immunity to verify whether the Anoscore could be used to predict the responsiveness of GI cancers to immunotherapy, thereby guiding individualized treatment. We assessed the TIME in the high- and low-Anoscore groups and found that immune cell abundance and immune checkpoint expression were significantly different between the two groups. The immune analysis revealed a surge in the infiltration of various immune cells, including B cells, DCs, macrophages, and neutrophils, in the high-Anoscore group. The infiltration of B cells and the formation of tertiary lymphoid structures are positively correlated with patients’ responses to immunotherapy (31–33). As vital sentinel cells, DCs play crucial roles in activating T cells and triggering antitumor immune responses (34). Macrophages and neutrophils exhibit dual functions in tumor immunity (35, 36). Our results suggested that immunotherapy may have better therapeutic effects on patients in the high-Anoscore group. Moreover, immunosuppressive receptors, such as CTLA4, ICOS, TIGIT, CD160 and TNFRSF9, and immunosuppressive ligands, such as CD274 (PD-L1), TNFSF9, ICOSLG, TNFSF18 and CD40LG, were highly expressed in the high-Anoscore group, which also suggested that the high-Anoscore group might react to immunotherapy more effectively.

Pharmacotherapy is a critical component of cancer treatment, and determining the drug sensitivity of tumors is important for guiding clinical therapy. Therefore, we analyzed drug sensitivity. Our analysis identified 141 drugs to which patients with high Anoscores were more sensitive, including chemotherapeutic agents, targeted therapies, and immunomodulatory drugs. Notably, cisplatin, paclitaxel, oxaliplatin, and 5-fluorouracil were classified as category 1 treatments for GI cancers, as recommended by the National Comprehensive Cancer Network (NCCN) Clinical Practice Guidelines (37–40). Patients in the low-Anoscore group were more sensitive to 24 types of drugs, most of which were novel small-molecule inhibitors, such as AMG-319, AZD2014, doramapimod and IGF1R_3801, than patients in the high-Anoscore group. The sensitivity analysis helped to distinguish to which drugs patients with different Anoscores were sensitive, thus facilitating the selection of clinical treatment drugs and promoting personalized treatment.

These results revealed the correlation between the Anoscore and tumor immunity, highlighting the potential value of the Anoscore in predicting the response of GI cancers to immunotherapy. We also analyzed drug sensitivity in GI cancers. In summary, identifying the Anoscore in patients with GI cancers is beneficial for selecting optimum drug treatments, including immunotherapy, chemotherapy and targeted therapy.

Anoscore, as the first prognostic model based on ARGs that integrated data from four major GI cancers (EC, GC, CC and RC) holds significant promise for clinical applications. This comprehensive approach enhanced its clinical applicability, providing a unified framework for risk stratification and prognostic evaluation across multiple GI cancer types. Firstly, Anoscore enables clinicians to more accurately identify high-risk patients. When combined with clinical characteristics, such as TNM staging, it can be incorporated into the Ano-nomogram to predict prognostic probabilities. Secondly, Anoscore serves as a valuable tool for both baseline assessments and dynamic monitoring during follow-up, facilitating timely adjustments to treatment strategies. Moreover, Anoscore can play a crucial role in identifying potential targets for immunotherapy, thereby broadening its application in personalized treatment planning. By elucidating the immune landscapes of GI tumors, Anoscore may assist clinicians in tailoring immunotherapy strategies to individual patients, optimizing treatment efficacy. In addition, our research demonstrated that patients in the high- and low-risk Anoscore groups exhibited distinct drug sensitivities. Anoscore has the potential to complement existing therapeutic regimens by guiding the selection of chemotherapy and targeted therapies. We anticipate that with continued research and refinement, Anoscore will enhance the scientific basis and precision of clinical decision-making. By integrating with current treatment paradigms, it is poised to advance the personalized medicine.

Despite the various methods used to establish our model, several limitations remain. The data used in this study were derived from public databases, which are limited in quantity and have incomplete clinicopathological information. Second, our study was a retrospective study. The above factors might contribute to biases in the construction of the Anoscore. Additionally, the mechanism by which anoikis affects the prognosis requires further research. Moreover, although we have conducted some validation experiments, the expression of some prognostic genes is still unclear, and how these genes affect the occurrence and development of GI tumors still needs further investigation.

Future research should focus on several critical areas to further establish the utility of Anoscore. Expanding the sample size and incorporating diverse patient populations will help assess the predictive performance of Anoscore across various cohorts. The roles and mechanisms of key genes in Anoscore, such as S100A11 and TLR3, in biological functions, including anoikis, tumor progression, immune modulation and drug sensitivity, warrant further comprehensive investigation through *in vitro* and *in vivo* experiments. Moreover, incorporating multi-omics data or integrating Anoscore with established biomarkers like PD-L1 and microsatellite instability (MSI) is one of the future research directions, which may enhance its predictive capacity and expand its clinical applications. Ultimately, further validation through prospective studies and clinical trials will be essential for assessing the real-world applicability of Anoscore in personalized medicine.

## 5 Conclusion

Overall, this study identified anoikis-associated molecular subgroups in GI cancers (ESCA, STAD, COAD, and READ) and developed a prognostic risk model based on ARGs, named Anoscore. The Anoscore performed effectively in predicting clinical outcomes for patients. Notably, the differences in the TIME and drug sensitivity between the high- and low-Anoscore groups highlighted its potential to distinguish cold and hot tumors and predict responses to drug therapies. Our findings are highly important for risk assessments and the personalized treatment of patients with GI cancers.

## Data Availability

The original contributions presented in the study are included in the article/**Supplementary Material**. Further inquiries can be directed to the corresponding authors.

## References

[1] Bray F Laversanne M Sung H Ferlay J Siegel RL Soerjomataram I. Global cancer statistics 2022: GLOBOCAN estimates of incidence and mortality worldwide for 36 cancers in 185 countries. CA: Cancer J Clin. (2024) 74:229–63. doi: 10.3322/caac.21834 38572751

[2] Huang Y Yin D Wu L. Identification of cuproptosis-related subtypes and development of a prognostic signature in colorectal cancer. Sci Rep. (2022) 12:17348. doi: 10.1038/s41598-022-22300-2 36253436 PMC9576756

[3] Ren Q Zhang P Zhang X Feng Y Li L Lin H. A fibroblast-associated signature predicts prognosis and immunotherapy in esophageal squamous cell cancer. Front Immunol. (2023) 14:1199040. doi: 10.3389/fimmu.2023.1199040 37313409 PMC10258351

[4] Liu Y Zheng H Gu AM Li Y Wang T Li C. Identification and validation of a metabolism-related prognostic signature associated with M2 macrophage infiltration in gastric cancer. Int J Mol Sci. (2023) 24:10625. doi: 10.3390/ijms241310625 37445803 PMC10342140

[5] Sattari Fard F Jalilzadeh N Mehdizadeh A Sajjadian F Velaei K. Understanding and targeting anoikis in metastasis for cancer therapies. Cell Biol Int. (2023) 47:683–98. doi: 10.1002/cbin.11970 36453448

[6] Yuan Z Li Y Zhang S Wang X Dou H Yu X. Extracellular matrix remodeling in tumor progression and immune escape: from mechanisms to treatments. Mol Cancer. (2023) 22:48. doi: 10.1186/s12943-023-01744-8 36906534 PMC10007858

[7] Chaojun L Pengping L Yanjun L Fangyuan Z Yaning H Yingbo S. TJP3 promotes T cell immunity escape and chemoresistance in breast cancer: a comprehensive analysis of anoikis-based prognosis prediction and drug sensitivity stratification. Aging (Albany NY). (2023) 15:12890–906. doi: 10.18632/aging.205208 PMC10713417 37950731

[8] Zhang L Zhu T Miao H Liang B. The calcium binding protein S100A11 and its roles in diseases. Front Cell Dev Biol. (2021) 9:693262. doi: 10.3389/fcell.2021.693262 34179021 PMC8226020

[9] Zheng X Li S Yang H. Roles of toll-like receptor 3 in human tumors. Front Immunol. (2021) 12:667454. doi: 10.3389/fimmu.2021.667454 33986756 PMC8111175

[10] Siegel RL Miller KD Fuchs HE Jemal A. Cancer statistics, 2022. CA Cancer J Clin. (2022) 72:7–33. doi: 10.3322/caac.21708 35020204

[11] Mitsui Y Tomonobu N Watanabe M Kinoshita R Sumardika IW Youyi C. Upregulation of mobility in pancreatic cancer cells by secreted S100A11 through activation of surrounding fibroblasts. Oncol Res. (2019) 27:945–56. doi: 10.3727/096504019X15555408784978 PMC7848232 31046874

[12] Sato H Sakaguchi M Yamamoto H Tomida S Aoe K Shien K. Therapeutic potential of targeting S100A11 in Malignant pleural mesothelioma. Oncogenesis. (2018) 7:11. doi: 10.1038/s41389-017-0017-3 29362358 PMC5833371

[13] Tu Y Xie P Du X Fan L Bao Z Sun G. S100A11 functions as novel oncogene in glioblastoma via S100A11/ANXA2/NF-κB positive feedback loop. J Cell Mol Med. (2019) 23:6907–18. doi: 10.1111/jcmm.v23.10 PMC6787445 31430050

[14] Koh SA Lee KH. HGF-mediated S100A11 overexpression enhances proliferation and invasion of gastric cancer. Am J Trans Res. (2018) 10:3385–94. PMC6291695 30662594

[15] Zheng M Meng H Li Y Shi J Han Y Zhao C. S100A11 promotes metastasis via AKT and ERK signaling pathways and has a diagnostic role in hepatocellular carcinoma. Int J Med Sci. (2023) 20:318–28. doi: 10.7150/ijms.80503 PMC9969497 36860671

[16] Niu Y Shao Z Wang H Yang J Zhang F Luo Y. LASP1-S100A11 axis promotes colorectal cancer aggressiveness by modulating TGFβ/Smad signaling. Sci Rep. (2016) 6:26112. doi: 10.1038/srep26112 27181092 PMC4867635

[17] Le Naour J Galluzzi L Zitvogel L Kroemer G Vacchelli E. Trial watch: TLR3 agonists in cancer therapy. Oncoimmunology. (2020) 9:1771143. doi: 10.1080/2162402X.2020.1771143 32934877 PMC7466857

[18] Castro FA Försti A Buch S Kalthoff H Krauss C Bauer M. TLR-3 polymorphism is an independent prognostic marker for stage II colorectal cancer. Eur J Cancer. (2011) 47:1203–10. doi: 10.1016/j.ejca.2010.12.011 21239167

[19] Chew V Tow C Huang C Bard-Chapeau E Copeland NG Jenkins NA. Toll-like receptor 3 expressing tumor parenchyma and infiltrating natural killer cells in hepatocellular carcinoma patients. J Natl Cancer Inst. (2012) 104:1796–807. doi: 10.1093/jnci/djs436 PMC3814220 23197495

[20] Yuan MM Xu YY Chen L Li XY Qin J Shen Y. TLR3 expression correlates with apoptosis, proliferation and angiogenesis in hepatocellular carcinoma and predicts prognosis. BMC Cancer. (2015) 15:245. doi: 10.1186/s12885-015-1262-5 25884709 PMC4435918

[21] Hsu WM Huang CC Wu PY Lee H Huang MC Tai MH. Toll-like receptor 3 expression inhibits cell invasion and migration and predicts a favorable prognosis in neuroblastoma. Cancer Lett. (2013) 336:338–46. doi: 10.1016/j.canlet.2013.03.024 23541683

[22] Smith M García-Martínez E Pitter MR Fucikova J Spisek R Zitvogel L. Trial Watch: Toll-like receptor agonists in cancer immunotherapy. Oncoimmunology. (2018) 7:e1526250. doi: 10.1080/2162402X.2018.1526250 30524908 PMC6279325

[23] Adams S. Toll-like receptor agonists in cancer therapy. Immunotherapy. (2009) 1:949–64. doi: 10.2217/imt.09.70 PMC2886992 20563267

[24] Aranda F Vacchelli E Obrist F Eggermont A Galon J Sautès-Fridman C. Trial Watch: Toll-like receptor agonists in oncological indications. Oncoimmunology. (2014) 3:e29179. doi: 10.4161/onci.29179 25083332 PMC4091055

[25] Devaud C John LB Westwood JA Darcy PK Kershaw MH. Immune modulation of the tumor microenvironment for enhancing cancer immunotherapy. Oncoimmunology. (2013) 2:e25961. doi: 10.4161/onci.25961 24083084 PMC3782527

[26] Zhao Q Yu J Meng X. A good start of immunotherapy in esophageal cancer. Cancer Med. (2019) 8:4519–26. doi: 10.1002/cam4.v8.10 PMC6712478 31231980

[27] Akin Telli T Bregni G Camera S Deleporte A Hendlisz A Sclafani F. PD-1 and PD-L1 inhibitors in oesophago-gastric cancers. Cancer Lett. (2020) 469:142–50. doi: 10.1016/j.canlet.2019.10.036 31669518

[28] Gambardella V Castillo J Tarazona N Gimeno-Valiente F Martínez-Ciarpaglini C Cabeza-Segura M. The role of tumor-associated macrophages in gastric cancer development and their potential as a therapeutic target. Cancer Treat Rev. (2020) 86:102015. doi: 10.1016/j.ctrv.2020.102015 32248000

[29] Lichtenstern CR Ngu RK Shalapour S Karin M. Immunotherapy, inflammation and colorectal cancer. Cells. (2020) 9:618. doi: 10.3390/cells9030618 32143413 PMC7140520

[30] Tolba MF. Revolutionizing the landscape of colorectal cancer treatment: The potential role of immune checkpoint inhibitors. Int J Cancer. (2020) 147:2996–3006. doi: 10.1002/ijc.v147.11 32415713

[31] Petitprez F de Reyniès A Keung EZ Chen TW Sun CM Calderaro J. B cells are associated with survival and immunotherapy response in sarcoma. Nature. (2020) 577:556–60. doi: 10.1038/s41586-019-1906-8 31942077

[32] Helmink BA Reddy SM Gao J Zhang S Basar R Thakur R. B cells and tertiary lymphoid structures promote immunotherapy response. Nature. (2020) 577:549–55. doi: 10.1038/s41586-019-1922-8 PMC8762581 31942075

[33] Cabrita R Lauss M Sanna A Donia M Skaarup Larsen M Mitra S. Tertiary lymphoid structures improve immunotherapy and survival in melanoma. Nature. (2020) 577:561–5. doi: 10.1038/s41586-019-1914-8 31942071

[34] Wculek SK Cueto FJ Mujal AM Melero I Krummel MF Sancho D. Dendritic cells in cancer immunology and immunotherapy. Nat Rev Immunol. (2020) 20:7–24. doi: 10.1038/s41577-019-0210-z 31467405

[35] Mantovani A Allavena P Marchesi F Garlanda C. Macrophages as tools and targets in cancer therapy. Nat Rev Drug Discovery. (2022) 21:799–820. doi: 10.1038/s41573-022-00520-5 35974096 PMC9380983

[36] Gungabeesoon J Gort-Freitas NA Kiss M Bolli E Messemaker M Siwicki M. A neutrophil response linked to tumor control in immunotherapy. Cell. (2023) 186:1448–64.e20. doi: 10.1016/j.cell.2023.02.032 37001504 PMC10132778

[37] Network NCC. NCCN Clinical Practice Guidelines in Oncology: Esophageal and Esophagogastric Junction Cancers, Version 5.2024 (2024). Available online at: www.nccn.org.

[38] Network NCC. NCCN Clinical Practice Guidelines in Oncology: Gastric Cancer, Version 5.2024 (2024). Available online at: www.nccn.org (Accessed January 8, 2025).

[39] Network NCC. NCCN Clinical Practice Guidelines in Oncology: Colon Cancer, Version 5.2024 (2024). Available online at: www.nccn.org (Accessed January 8, 2025).

[40] Network NCC. NCCN Clinical Practice Guidelines in Oncology: Rectal Cancer, Version 4.2024 (2024). Available online at: www.nccn.org (Accessed January 8, 2025).

