# Supplementary material for: An anoikis-related signature predicts prognosis and immunotherapy response in gastrointestinal cancers
Source: Front Immunol. 2025 Feb 6;16:1477913. doi: 10.3389/fimmu.2025.1477913 (PMC11839610; doi:10.3389/fimmu.2025.1477913)
Supplement: Supplementary file 1 [file DataSheet1.pdf]

Additional File 1

Supplementary Table S1. Sequences of the siRNAs.

S100A11-siRNA1	GAAAGGATGGTTATAACTA
S100A11-siRNA2	CTTCATGAATACAGAACTA
S100A11-siRNA3	GTCCTTGACCGCATGATGA

Supplementary Table S2. The sequences of primers used for qRT–PCR.

	Primer sequences
S100A11-F	TCTCCAAGACAGAGTTCCTAAGC
S100A11-R	CTGTTGGTGTCCAGTTTCTTCATC
TLR3-F	CCTGATGAAATGTCTGGATTTGGA
TLR3-R	AACAGTGCACTTGGTGGTGGAG
GAPDH-F	GCACCGTCAAGGCTGAGAAC
GAPDH-R	TGGTGAAGACGCCAGTGGA

Supplementary Table S3. ARGs that constitute the Anoscore and their corresponding coefficients.

	Gene	Coefficient
1	PDK4	0.179
2	CDKN2A	0.078
3	MYC	-0.180
4	FGF2	0.130
5	TLR3	-0.291
6	IL1RAP	0.124
7	CD36	0.120
8	TRAF2	-0.222
9	TNFRSF12A	0.145
10	NOTCH3	0.134
11	S100A11	0.260
12	WNT2	-0.109

Supplementary Table S4. Drugs with differential sensitivity in low- and high-Anoscore subgroups.

Drug	Sensitive group (based on the Anoscore)	*p*
5-Fluorouracil	high	$< 2.22e^{-16}$
ABT737	high	$3.3e^{-05}$
Acetalax	high	$< 2.22e^{-16}$

Afatinib	high	2.8 e^{-09}
Afuresertib	high	< 2.22e^{-16}
AGI-5198	high	< 2.22e^{-16}
AGI-6780	high	< 2.22e^{-16}
Alisertib	high	8.1e^{-05}
AMG-319	low	0.00063
AT13148	high	< 2.22e^{-16}
Axitinib	high	< 2.22e^{-16}
AZ960	low	2.7e^{-11}
AZ6102	high	< 2.22e^{-16}
AZD1208	high	9.4e^{-11}
AZD1332	low	5e^{-14}
AZD2014	low	7.5e^{-11}
AZD3759	high	< 2.22e^{-16}
AZD4547	high	8.5e^{-08}
AZD5153	high	< 2.22e^{-16}
AZD5363	high	0.00044
AZD5438	high	< 2.22e^{-16}
AZD5582	low	< 2.22e^{-16}
AZD5991	high	< 2.22e^{-16}
AZD6738	high	< 2.22e^{-16}
AZD7762	high	1.3e^{-15}
AZD8055	low	< 2.22e^{-16}
AZD8186	low	< 2.22e^{-16}
BDP-00009066	high	4.6e^{-16}
BIBR-1532	high	< 2.22e^{-16}
BMS-345541	high	< 2.22e^{-16}
BMS-536924	high	< 2.22e^{-16}
BMS-754807	low	< 2.22e^{-16}
Bortezomib	high	< 2.22e^{-16}
BPD-00008900	high	4.3e^{-12}
Buparlisib	high	7.1e^{-14}
Camptothecin	high	< 2.22e^{-16}
Carmustine	high	< 2.22e^{-16}
CDK9_5038	low	< 2.22e^{-16}
CDK9_5576	high	4.7e^{-08}
Cediranib	high	3.9e^{-05}
Cisplatin	high	< 2.22e^{-16}
Crizotinib	high	< 2.22e^{-16}
Cyclophosphamide	high	< 2.22e^{-16}
Cytarabine	high	< 2.22e^{-16}
Dabrafenib	high	< 2.22e^{-16}

Dactinomycin	high	$1.6e^{-12}$
Dactolisib	high	$1.6e^{-12}$
Daporinad	high	$< 2.22e^{-16}$
Dasatinib	low	$< 2.22e^{-16}$
Dihydrorotenone	high	$< 2.22e^{-16}$
Dinaciclib	high	$8.5e^{-09}$
Docetaxel	high	$< 2.22e^{-16}$
Doramapimod	low	$< 2.22e^{-16}$
Eg5_9814	high	$< 2.22e^{-16}$
Elephantin	high	$< 2.22e^{-16}$
Entinostat	high	$5.4e^{-07}$
Epirubicin	high	0.00052
EPZ004777	high	$< 2.22e^{-16}$
EPZ5676	high	$< 2.22e^{-16}$
ERK_2440	high	$4.3e^{-11}$
ERK_6604	high	$4.4e^{-13}$
Erlotinib	high	$< 2.22e^{-16}$
Fludarabine	high	$2e^{-08}$
Foretinib	high	$< 2.22e^{-16}$
Fulvestrant	high	$< 2.22e^{-16}$
Gallibiscoquinazole	high	$< 2.22e^{-16}$
GDC0810	high	$< 2.22e^{-16}$
Gefitinib	high	$< 2.22e^{-16}$
Gemcitabine	high	$< 2.22e^{-16}$
GSK343	high	$< 2.22e^{-16}$
GSK591	high	$< 2.22e^{-16}$
GSK269962A	high	$9.2e^{-09}$
GSK1904529A	high	$< 2.22e^{-16}$
GSK2578215A	high	$< 2.22e^{-16}$
GSK2606414	high	0.00083
IAP_5620	high	$< 2.22e^{-16}$
I-BET-762	high	$< 2.22e^{-16}$
I-BRD9	high	$< 2.22e^{-16}$
IGF1R_3801	low	0.00017
Ipatasertib	high	$< 2.22e^{-16}$
IRAK4_4710	high	$< 2.22e^{-16}$
Irinotecan	high	$1.4e^{-13}$
IWP-2	high	$1.2e^{-05}$
JAK_8517	low	$< 2.22e^{-16}$
JQ1	low	$< 2.22e^{-16}$
KRAS (G12C) Inhibitor-12	high	$< 2.22e^{-16}$
KU-55933	low	$7.8e^{-05}$

Lapatinib	high	$< 2.22e^{-16}$
LCL161	high	$< 2.22e^{-16}$
Leflunomide	high	$< 2.22e^{-16}$
LGK974	high	$< 2.22e^{-16}$
Linsitinib	high	$< 2.22e^{-16}$
LJI308	high	$7.3e^{-10}$
MG-132	high	$< 2.22e^{-16}$
MIM1	high	$< 2.22e^{-16}$
MIRA-1	high	$< 2.22e^{-16}$
Mirin	high	$3.5e^{-07}$
Mitoxantrone	low	$1.6e^{-05}$
MK-1775	high	$< 2.22e^{-16}$
MK-2206	high	$9.3e^{-06}$
MK-8776	high	$8.5e^{-07}$
ML323	high	$< 2.22e^{-16}$
MN-64	high	0.00083
Navitoclax	high	$< 2.22e^{-16}$
Nelarabine	high	$5.8e^{-11}$
Nilotinib	high	$< 2.22e^{-16}$
NU7441	low	$< 2.22e^{-16}$
NVP-ADW742	high	$< 2.22e^{-16}$
Obatoclax Mesylate	high	$< 2.22e^{-16}$
OF-1	high	$< 2.22e^{-16}$
OSI-027	high	$< 2.22e^{-16}$
Osimertinib	high	$9.2e^{-11}$
OTX015	high	$6.2e^{-11}$
Oxaliplatin	high	$< 2.22e^{-16}$
P22077	high	$< 2.22e^{-16}$
Paclitaxel	high	$< 2.22e^{-16}$
PAK_5339	high	$6.9e^{-11}$
Palbociclib	high	$< 2.22e^{-16}$
PD173074	high	$3e^{-05}$
PD0325901	high	$< 2.22e^{-16}$
Pevonedistat	high	$< 2.22e^{-16}$
PF-4708671	high	$< 2.22e^{-16}$
PFI3	high	$4.8e^{-05}$
Picolinici-acid	high	$< 2.22e^{-16}$
PLX-4720	high	$1.4e^{-05}$
Podophyllotoxin bromide	high	$< 2.22e^{-16}$
PRT062607	low	$4.9e^{-14}$
Rapamycin	high	$< 2.22e^{-16}$
Ribociclib	high	$6.8e^{-07}$

Ruxolitinib	high	$7.4\ e^{-08}$
Sapitinib	high	$4e^{-05}$
Savolitinib	high	$< 2.22e^{-16}$
SB216763	low	$6.9\ e^{-05}$
SCH772984	high	$< 2.22e^{-16}$
Selumetinib	high	$6.2e^{-09}$
Sepantronium bromide	high	$1.1e^{-07}$
Sinularin	high	$< 2.22e^{-16}$
Sorafenib	high	$< 2.22e^{-16}$
Staurosporine	low	$7.8e^{-06}$
TAF1_5496	high	$< 2.22e^{-16}$
Talazoparib	high	$< 2.22e^{-16}$
Tamoxifen	high	$< 2.22e^{-16}$
Telomerase Inhibitor IX	high	$< 2.22e^{-16}$
Temozolomide	high	$< 2.22e^{-16}$
Tozasertib	high	$< 2.22e^{-16}$
Trametinib	high	$< 2.22e^{-16}$
Ulixertinib	high	$< 2.22e^{-16}$
ULK1_4989	low	0.00045
VE821	high	$< 2.22e^{-16}$
VE-822	high	$8.1e^{-09}$
Venetoclax	high	$8e^{-1}3$
Vinblastine	high	$< 2.22e^{-16}$
Vincristine	high	$1.6e^{-11}$
Vinorelbine	high	$< 2.22e^{-16}$
Vorinostat	high	$< 2.22e^{-16}$
VSP34_8731	high	$< 2.22e^{-16}$
VX-11e	high	$< 2.22e^{-16}$
Wee1 Inhibitor	high	$< 2.22e^{-16}$
WEHI-539	high	$2.7e^{-13}$
Wnt-C59	high	$4.6e^{-09}$
WZ4003	low	$< 2.22e^{-16}$
XAV939	low	$< 2.22e^{-16}$
YK-4-279	high	$< 2.22e^{-16}$
ZM447439	low	$2e^{-06}$
Zoledronate	high	$< 2.22e^{-16}$
