# Supplementary material for: An anoikis-related signature predicts prognosis and immunotherapy response in gastrointestinal cancers
Source: Front Immunol. 2025 Feb 6;16:1477913. doi: 10.3389/fimmu.2025.1477913 (PMC11839610; doi:10.3389/fimmu.2025.1477913)
Supplement: Supplementary file 2 [file DataSheet2.pdf]

Additional File 1

Supplementary Fig. S1

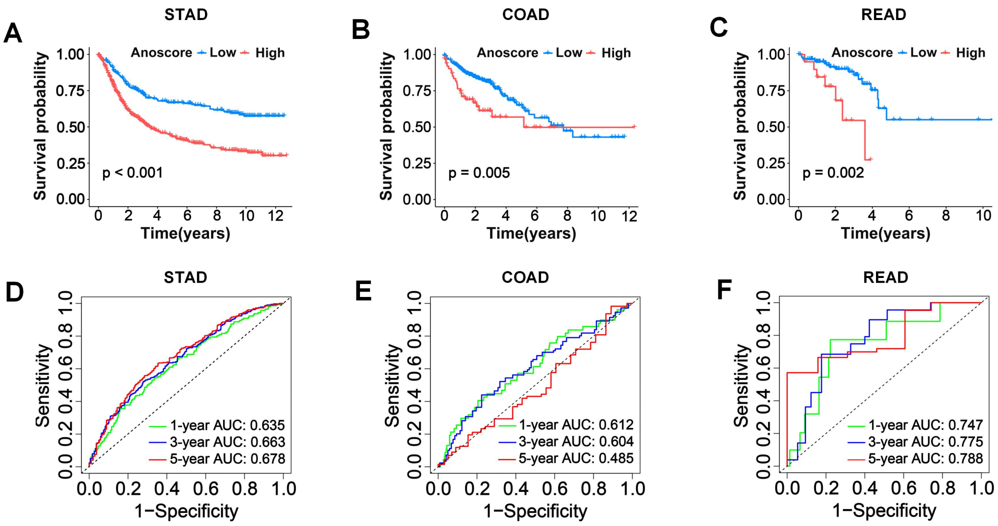

Supplementary Figure S1. A-C KM analysis of the Anoscore in patients with STAD **(A)**, COAD **(B)** and READ **(C)**. **D-F** ROC curves based on the Anoscore for patients with STAD **(D)**, COAD **(E)** and READ **(F)**.

Supplementary Fig. S2

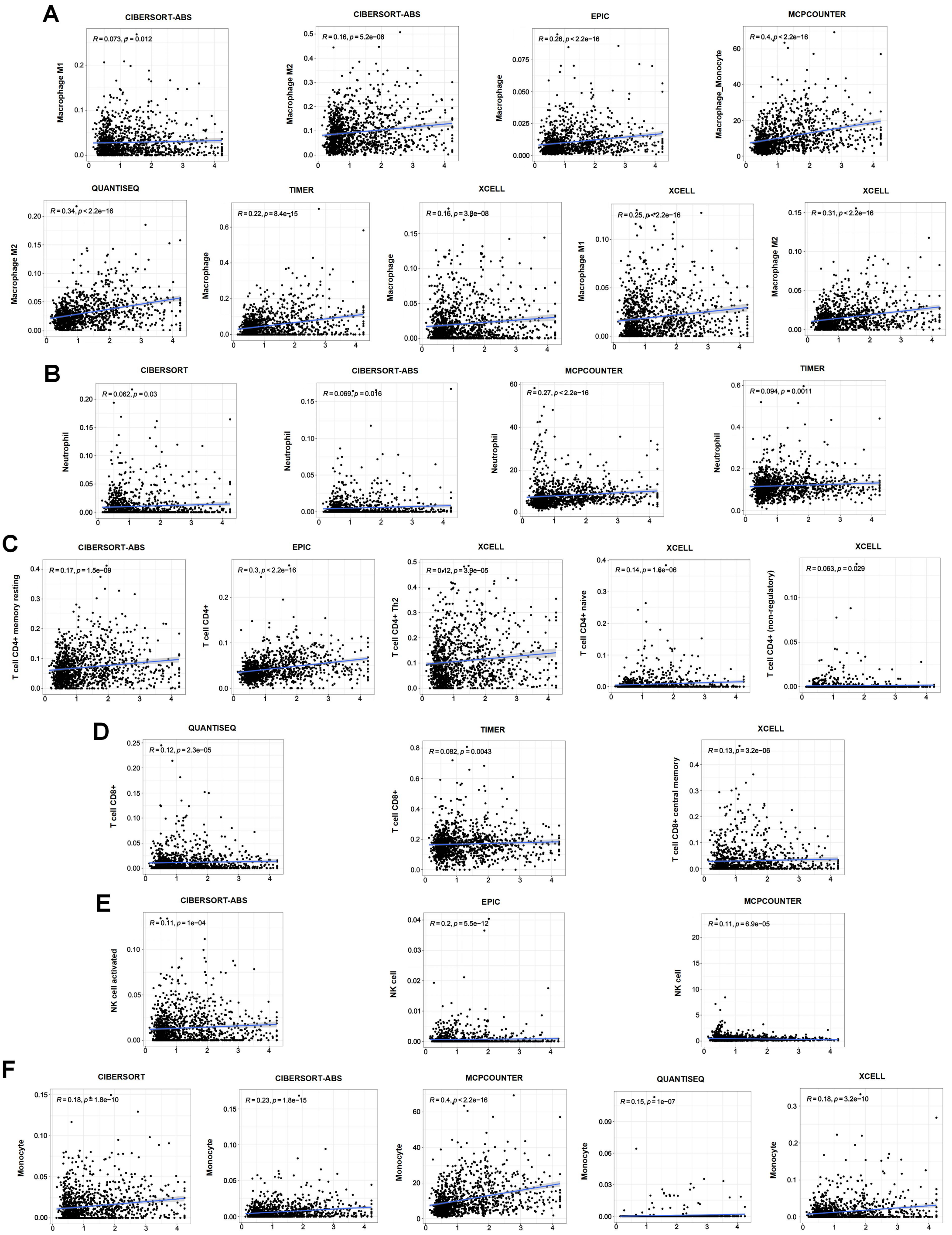

Figure S2. Correlation analysis between immune cell infiltration and the Anoscore. **A** Associations between macrophages and the Anoscore using CIBERSORT-ABS, EPIC, MCPCOUNTER, QUANTISEQ, TIMER and XCELL. **B** Associations between neutrophils and the Anoscore using CIBERSORT, CIBERSORT-ABS, MCPCOUNTER and TIMER. **C**

Associations between $CD4^+$ T cells and the Anoscore using CIBERSORT-ABS, EPIC and XCELL. **D** Associations between $CD8^+$ T cells and the Anoscore using QUANTISEQ, TIMER and XCELL. **E** Associations between NK cells and the Anoscore using QUANTISEQ, TIMER and XCELL. **F** The associations between monocytes and the Anoscore using CIBERSORT, CIBERSORT-ABS, MCPCOUNTER, QUANTISEQ and XCELL.

Supplementary Fig. S3

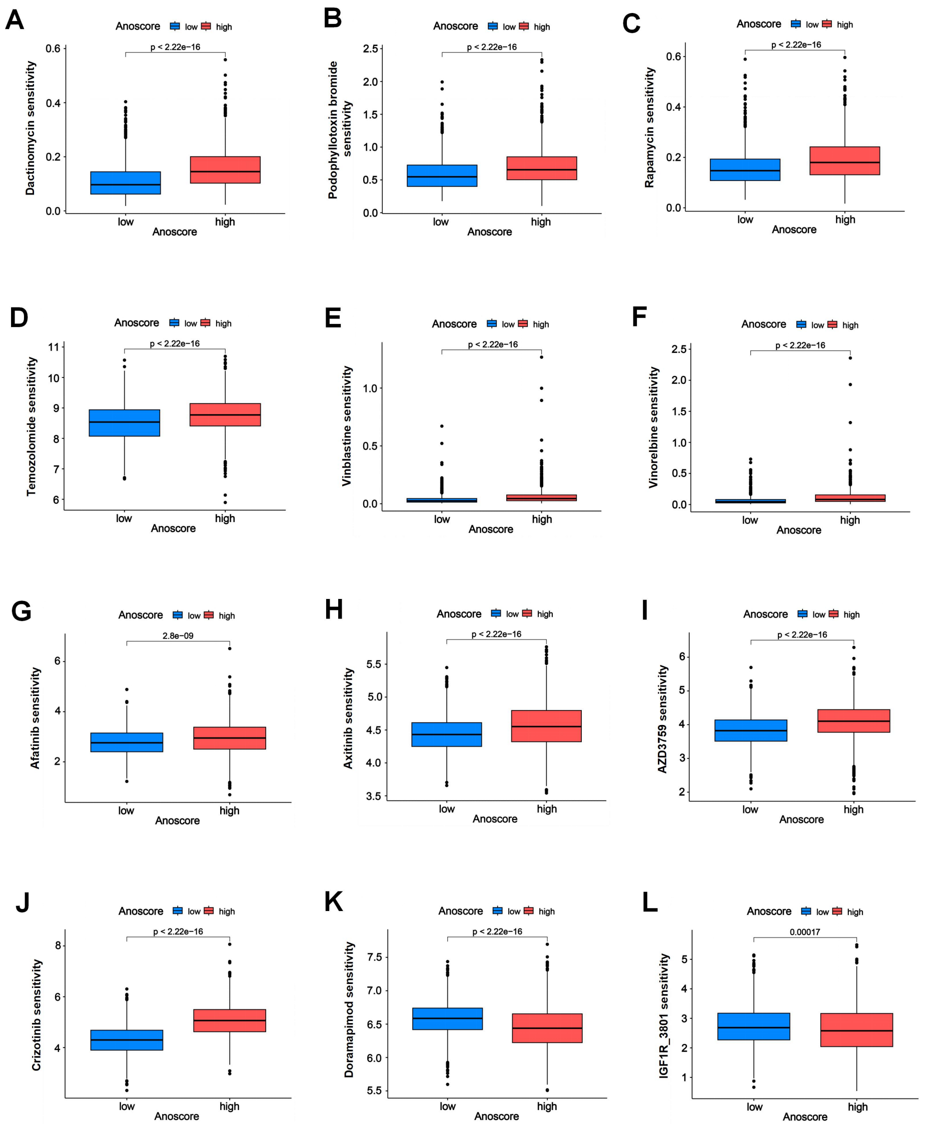

Figure S3. Drug sensitivity in the low- and high-Anoscore groups. **A-G** Analysis of the sensitivity of the high- and low-Anoscore groups to chemotherapy drugs, including dactinomycin **(A)**, podophyllotoxin **(B)**, rapamycin **(C)**, temozolomide **(D)**, vinblastine **(E)**, vinorelbine **(F)** and dactinomycin **(G)**. **H-L** Analysis of the sensitivity of the high- and low-Anoscore groups to targeted drugs, including afatinib **(H)**, axitinib **(I)**, AZD3759 **(J**, an mTOR inhibitor**)**, crizotinib **(K)** and IGF1R-3801 **(L**, an IGF1R inhibitor**)**.

Supplementary Fig. S4

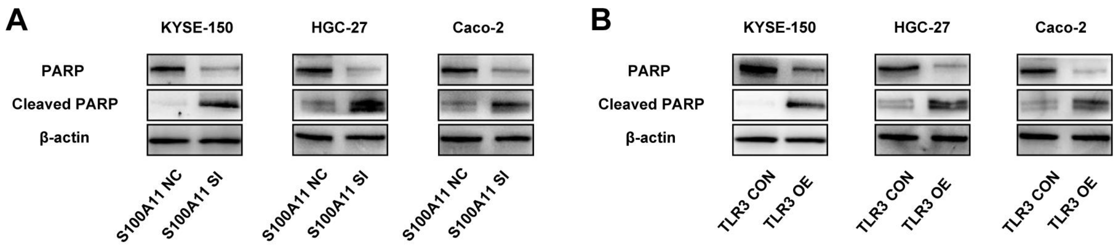

Figure S4. The expression of PARP and cleaved PARP in S100A11-depleted **(A)** and TLR3-overexpressing **(B)** cells by WB.
